# Discovery of recessive effect of human polymerase δ proofreading deficiency through mutational analysis of POLD1-mutated normal and cancer cells

**DOI:** 10.1038/s41431-024-01598-8

**Published:** 2024-04-24

**Authors:** Maria A. Andrianova, Vladimir B. Seplyarskiy, Mariona Terradas, Ana Beatriz Sánchez-Heras, Pilar Mur, José Luis Soto, Gemma Aiza, Emma Borràs, Fyodor A. Kondrashov, Alexey S. Kondrashov, Georgii A. Bazykin, Laura Valle

**Affiliations:** 1 Institute for Research in Biomedicine (IRB Barcelona), The Barcelona Institute of Science and Technology, Barcelona, Spain; 2 Department of Biomedical Informatics, Harvard Medical School, Boston, MA USA; 3 Division of Genetics, Brigham and Women’s Hospital, Harvard Medical School, Boston, MA USA; 4 Hereditary Cancer Program, Catalan Institute of Oncology, Oncobell Program, IDIBELL, Hospitalet de Llobregat, Barcelona, Spain; 5 Foundation for the Promotion of Health and Biomedical Research of Valencia Region (FISABIO), Elche Health Department, Elche, Spain; 6 Medical Oncology Department, Cancer Genetic Counseling Unit. Elche University Hospital, Elche, Spain; 7 Department of Health of Catalonia, Catalan Cancer Plan, Barcelona, Spain; 8 Molecular Genetics Unit, Elche University Hospital, Elche, Spain; 9 Molecular Genetics Unit, Consorci Sanitari de Terrassa, Terrassa, Spain; 10 Institute of Science and Technology Austria, Klosterneuburg, Austria; 11 Evolutionary and Synthetic Biology Unit, Okinawa Institute of Science and Technology Graduate University, Okinawa, Japan; 12 Department of Ecology and Evolutionary Biology, University of Michigan, Ann Arbor, MI USA; 13 Centro de Investigación Biomédica en Red de Cáncer (CIBERONC), Madrid, Spain

**Keywords:** Cancer genomics, Cancer genomics, Mutation

## Abstract

Constitutional heterozygous pathogenic variants in the exonuclease domain of *POLE* and *POLD1*, which affect the proofreading activity of the corresponding polymerases, cause a cancer predisposition syndrome characterized by increased risk of gastrointestinal polyposis, colorectal cancer, endometrial cancer and other tumor types. The generally accepted explanation for the connection between the disruption of the proofreading activity of polymerases epsilon and delta and cancer development is through an increase in the somatic mutation rate. Here we studied an extended family with multiple members heterozygous for the pathogenic *POLD1* variant c.1421T>C p.(Leu474Pro), which segregates with the polyposis and cancer phenotypes. Through the analysis of mutational patterns of patient-derived fibroblasts colonies and de novo mutations obtained by parent-offspring comparisons, we concluded that heterozygous *POLD1* L474P just subtly increases the somatic and germline mutation burden. In contrast, tumors developed in individuals with a heterozygous mutation in the exonuclease domain of *POLD1*, including L474P, have an extremely high mutation rate (>100 mut/Mb) associated with signature SBS10d. We solved this contradiction through the observation that tumorigenesis involves somatic inactivation of the wildtype *POLD1* allele. These results imply that exonuclease deficiency of polymerase delta has a recessive effect on mutation rate.

## Introduction

Disruption of DNA repair is one of the major mechanisms underlying hereditary cancer. Like classic tumor suppressor genes, inactivation of DNA repair genes usually follows the Knudson’s two-hit hypothesis, which requires inactivation of the two alleles of the gene (e.g. cancer-associated MMR genes, *MUTYH*, *BRCA1* and *BRCA2*, among others) to disrupt the specific DNA repair mechanism [1, 2]. In particular, for autosomal dominant cancer syndromes, where one allele is constitutionally mutated, a somatic mutation that disrupts the unaffected copy of the gene in the target tissue is required [2]. The two-hit model in the context of DNA repair makes intuitive sense, with one functional copy of the gene being sufficient to preserve genome maintenance. However, variants disrupting the exonuclease activity of major DNA replication polymerases (epsilon and delta) were proposed to be an exception: alteration of one exonuclease allele might be enough to ramp up the error rate in the DNA synthesized by the mutated protein [3]. Proofreading deficiency of the corresponding polymerases causes an autosomal dominant cancer predisposition syndrome called polymerase proofreading-associated polyposis (PPAP). Since the description of the syndrome, the haploinsufficiency hypothesis has been supported by the lack of somatic mutation or loss of heterozygosity identified in many PPAP-associated tumors; observation mainly based on data derived from *POLE*-associated PPAP cancers and adenomas [3–5]. However, recent evidence has shown that some pathogenic heterozygous variants identified in PPAP patients, specifically variants in *POLD1*, have a weak effect on mutation rate in somatic tissues [6], raising doubts about the validity of the haploinsufficiency hypothesis for this particular gene. Moreover, previous studies in yeast and murine models suggest a recessive effect for Polδ proofreading inactivation, both in terms of mutation rate and phenotype [7–11].

To shed light on this conundrum, we studied an extended family with multiple members heterozygous for the *POLD1* pathogenic variant c.1421T>C; p.(Leu474Pro), further referred to as *POLD1* L474P. In the family, most *POLD1* L474P carriers had been diagnosed with multiple colorectal polyps, colorectal cancer, and/or endometrial cancer. We sequenced the genomes of seven *POLD1* L474P carriers and five *POLD1* wildtype family members. We also analyzed the exomes or genomes of the tumors developed by three individuals with different constitutional *POLD1* pathogenic variants (L474P, D316H and S478N). We evaluated the genomic sequences obtained from single cell-derived fibroblasts’ colonies cultured for ~40 passages from multiple *POLD1* L474P positive and negative members of the family under study. With this set-up, we were able to estimate the somatic mutagenic effect of heterozygous *POLD1* L474P in a controlled experiment, and we complemented this study with the detection of de novo germline mutations using a robust methodology. Overall, we found that the heterozygous *POLD1* L474P increases the mutation rate by less than 15% in both soma and germline, while in the associated cancers, somatic loss of the wildtype copy of *POLD1* leads to a dramatic increase in mutation rate.

## Material and methods

The methodology used in this study is detailed in a supplementary file (Supplementary Material and Methods). The study received the approval of the Ethics Committees of IDIBELL (PR235/16) and the University Hospital of Elche (PI37/2018).

## Results

### Characteristics of the *POLD1* c.1421T>C (p.Leu474Pro) variant and of the family under study

*POLD1* c.1421T>C (p.Leu474Pro) is a missense variant located in the Exo IV motif of the exonuclease domain of *POLD1*, affecting a residue of the exonuclease that is in direct contact with the DNA (at less than 6 Å). In silico tools assign strong pathogenicity predictions to this variant (REVEL score: 0.913; 1 being the highest score in favor of pathogenicity). While it has not been reported in gnomAD datasets (v.2.1.1, v.3.1.2 and v.4.0.0), L474P has been recurrently identified in families with PPAP-suggestive phenotypes, where co-segregation with the tumor phenotypes was observed [4, 5, 12]. A mutator phenotype was detected when the homologous residue was substituted (Leucine to Serine) in the haploid strain of *Saccharomyces cerevisiae* [13], and functional results obtained in this study showed that the substitution of the wildtype residue (Leu) to proline (Pro), the amino acid identified in the patients, also cause a mutator phenotype in a haploid strain of *Schizosaccharomyces pombe* (Supplementary Fig. S1). Supported by available evidence, *POLD1* L474P may be classified as pathogenic following the ACMG/AMP guidelines (Supplementary Note 1) [14].

**Fig. 1:**
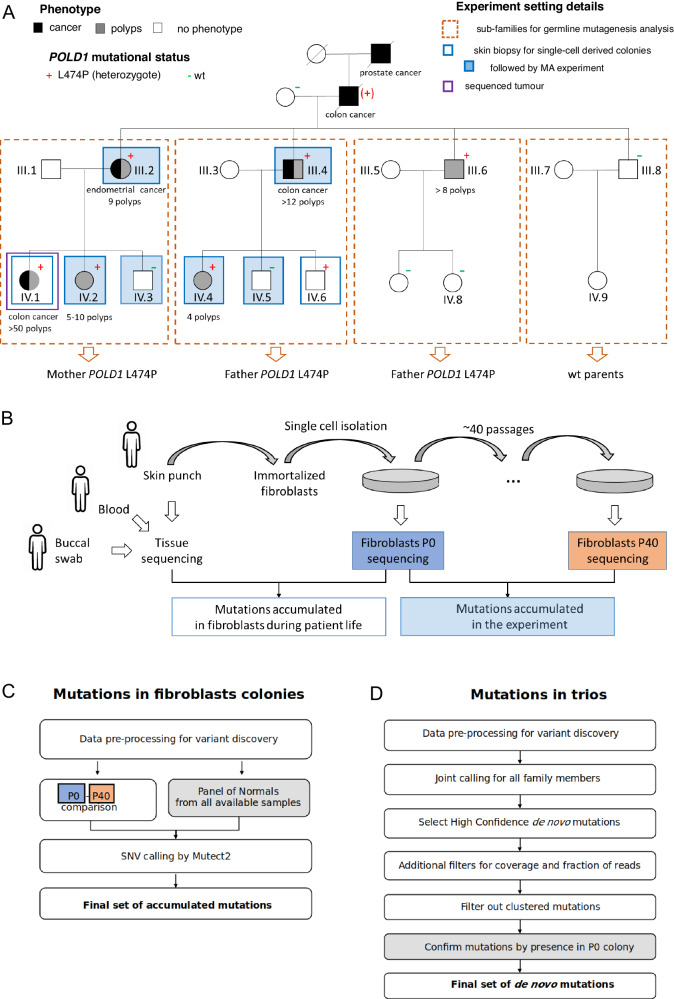
Family, sample description, and study pipeline. **A** Family pedigree. Individuals diagnosed with cancer are marked in black, and with polyps, in gray. The tumor types and number of polyps are indicated below the corresponding individual symbol. Detailed tumor phenotypes and ages at diagnosis are shown in Supplementary Table S1. Blue rectangles mark the individuals from whom skin biopsies were obtained for the culture of single-cell fibroblast colonies. Filled blue rectangles depict the individuals whose single-cell fibroblast colonies were used for the mutation accumulation experiments. The violet rectangle highlights the individual with a sequenced tumor sample. Sequenced sub-families are framed by rectangles made with dashed orange lines, where individual IDs are detailed for the sequenced individuals. Plus and minus signs mark the *POLD1* L474P carrier status, and a plus sign between parentheses (+) indicates an obligate carrier. **B** Experimental workflow performed on immortalized fibroblasts from family members for the assessment of somatic mutation accumulation. **C** Algorithm used for calling somatic mutations accumulated during cell line growth. **D** Algorithm used for calling germline de novo mutations.

In the family investigated in this study, *POLD1* L474P was identified in seven members (plus one obligated carrier), three of whom had been diagnosed with early-onset colorectal cancer (ages at cancer diagnosis: 23–50), and one with endometrial cancer at age 58. Most L474P carriers were diagnosed with gastrointestinal polyps, mainly with mild polyposis phenotypes (Fig. 1A, Supplementary Table S1).

### Influence of constitutional *POLD1* L474P on somatic mutagenesis

To study the effect of *POLD1* L474P on somatic mutation rate, we designed a controlled experiment in six cell lines obtained from fibroblasts (origin: skin punches) of individuals III.2, IV.2, IV.3, III.4, IV.4 and IV.5, which included four L474P heterozygotes and two wildtype family members (Fig. 1A, filled blue rectangles). For each individual, a single cell-derived fibroblast colony was sequenced at the initial timepoint (short after single cell isolation). The six cell lines were grown for ~40 passages (Supplementary Table S2), and genome sequencing was performed on the DNA obtained from the cultured cells (experiment endpoint) (Fig. 1B, Supplementary Materials and Methods). The detection of mutations accumulated during the passages was performed using a standard method for somatic single nucleotide variant (SNV) calling (Fig. 1C, Supplementary Materials and Methods).

**Fig. 2:**
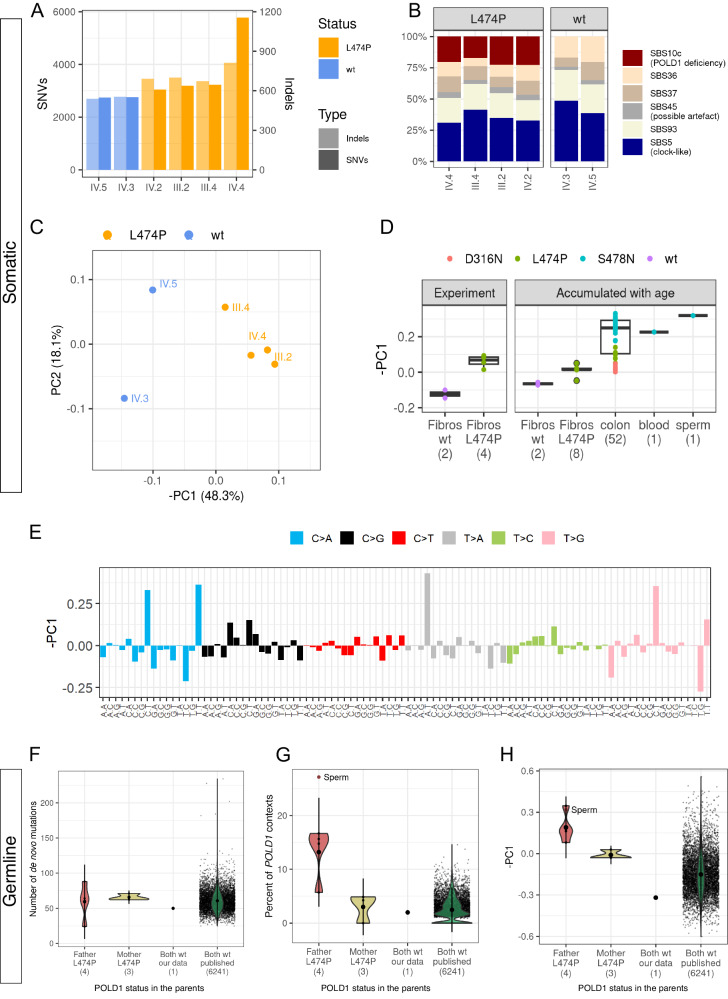
Somatic and germline mutation burden and spectrum. **A** Number of SNVs and indels accumulated during the experiment in the cultured fibroblasts of carriers and non-carriers of *POLD1* L474P. **B** Proportion of mutations attributed to COSMIC mutational signatures accumulated in the cultured fibroblasts. De novo signatures were extracted and decomposed to COSMIC signatures using SigProfilerExtractor with subsequent refitting using SigFit. **C** PCA analysis of 96-context mutational spectra of mutations accumulated in the cultured fibroblasts. **D** Values of the PC1 component in wildtype (wt) and L474P fibroblast colonies obtained in the mutation accumulation experiment, as well as PC1 component values obtained from the sequence analysis of other normal tissues obtained from heterozygous carriers of *POLD1* pathogenic variants: skin fibroblasts (sequenced in this study) and other tissues^6^. The different colors correspond to different *POLD1* pathogenic variants. **E** Loadings of mutational contexts in –PC1. **F** Number of de novo mutations in the offspring of parents with wildtype or mutated *POLD1*. **G** Proportion of mutated *POLD1*-specific tri-nucleotide contexts in de novo mutations in offspring of parents with wildtype or mutated *POLD1*. **H** Value of -PC1 in de novo mutations in offspring of parents with wildtype or mutated *POLD1*. In (**G**) and (**H**), the red dot (sperm) corresponds to the sperm sample from an individual with constitutional *POLD1* S478N previously reported^6^. In (**D**), and (**F**–**H**), numbers between parentheses indicate the number of analyzed samples.

While the number of mutations accumulated during the experiment in fibroblasts harboring *POLD1* L474P was higher than in wildtype fibroblasts, for most samples this increase was relatively minor (Fig. 2A). For three out of four carriers (III.2, III.4, IV.2) the effect was slightly stronger for indels than for SNVs, while the opposite was observed in the cell line derived from the cultured fibroblasts of IV.4 (Fig. 2A). Single T insertions in homopolymer tracts were enriched in *POLD1* L474P heterozygotes compared to wildtype colonies, agreeing with previous results obtained from the sequencing of normal tissues of individuals heterozygous for *POLD1* pathogenic variants [6]. This pattern corresponds to COSMIC signature ID1 (Supplementary Fig. S2). Although the effect on the overall mutation rate was small, *POLD1* L474P heterozygous carrier status was associated with a significant shift in the mutational spectrum. De novo extraction and decomposition of mutational signatures in the endpoint fibroblast cell lines derived from the four carriers and the two non-carriers of *POLD1* L474P yielded six distinct COSMIC mutational signatures. Refitting the mutational spectra to this fixed number of signatures in each sample revealed a highly significant presence of the Polδ proofreading deficiency signature SBS10c in all four *POLD1* L474P heterozygotes but not in the wildtype cell lines (Fig. 2B, Supplementary Table S3). The excess of mutations in *POLD1* L474P fibroblasts was mostly due to SBS10c (Supplementary Fig. S3).

To orthogonally assess the effect of *POLD1* L474P on mutational spectrum, we calculated the 96-context mutational spectra for the fibroblasts with and without *POLD1* L474P and applied principal component analysis (PCA) to these spectra (Fig. 2C). PCA clearly separated L474P carriers from non-carriers: PC1 explained ~48% of the variance in the mutational spectrum across samples and reflected the experimentally obtained difference in spectra between *POLD1* mutated and non-mutated individuals (Fig. 2D, E). Comparison of the principal components with COSMIC mutational signatures showed that PC1 had the strongest correlation with SBS10c (cosine similarity = 0.52). PC1 also separated previously published samples of somatic tissues (colon, blood, sperm) from individuals with constitutional pathogenic variants in *POLD1* (Fig. 2D). Moreover, the obtained PC1 values differed among samples with different pathogenic variants in *POLD1*: S478N showed the highest PC1 values, and D316N, the lowest. This is consistent with previous findings that showed that *POLD1* S478N had the strongest effect on mutational burden, while this effect was much lower for D316N [6].

Similar to a previous study [6], we searched for the footprint of polymerase delta (Polδ) proofreading deficiency in the mutations accumulated in tissues over the lifetime of the studied individuals (Supplementary Note 2). The signal of Polδ proofreading deficiency was also present in these data, however, the separation from non-carriers was fuzzier compared to the experimental data (Fig. 2D).

### Influence of constitutional *POLD1* L474P on germline mutagenesis

To study the impact of *POLD1* L474P on mutation patterns in the germline, we sequenced four subfamilies from the extended family, each including two parents and one to three children, comprising a total of eight offspring (Fig. 1A, Supplementary Table S1). In two subfamilies, *POLD1* L474P was inherited from the father; in one subfamily, from the mother; and in the remaining subfamily, both parents had wildtype *POLD1*. De novo mutations in each offspring were called using the standard GATK4 pipeline with additional filters (Supplementary Material and Methods), focused on identifying mutations present in the offspring but absent in both parents (Fig. 1D). The number of called de novo mutations per individual varied between 24 and 88 (Fig. 2F).

Theoretically, *POLD1* L474P should affect the number of replication errors. However, based on the obtained data from the fibroblasts sequencing experiment, we expected this effect to be minor, with a ~15% increase in the overall number of mutations in the offspring due to the heterozygous *POLD1* L474P genotype. Because of the ~10-fold higher number of cell divisions in spermatocytes compared to oocytes [15], one would also expect a larger increase in mutation rate in male gametes. However, our (limited) data showed no significant effect of heterozygous *POLD1* L474P on the burden of de novo mutations, when either the father or the mother carried the variant (Fig. 2F). We next performed an analysis of mutational spectra, which would be better powered because *POLD1* L474P produces mutations in very specific contexts and its spectrum is well described by four main mutation types: CpCpT>A, TpCpT>A, ApTpT>A and CpTpT>G (Fig. 2E). The proportion of de novo mutations in these contexts among all 96 contexts estimated from previously published trios [16, 17] was ~2.4%. Given this and the total number of observed de novo mutations per offspring of fathers with *POLD1* L474P, we expected a total of 5.7 mutations in these contexts for four offspring. Instead, we observed 28 of such mutations (which correspond to 11.8% of all mutations identified), suggesting a ~4.9-fold increase (rate ratio test *p*-value = 6.62 × 10^−5^, Fig. 2G). These observations indicate that in spermatocytes, the fraction of mutations caused by replicative errors introduced by heterozygous *POLD1* L474P is comparable to that observed in fibroblasts (14.7% in soma and 11.8% in germline). By contrast, in the offspring of *POLD1* L474P mothers, the fraction of the associated mutations was practically the same as in the wildtype trios (Fig. 2G, Supplementary Fig. S4). Consistently, the projection of the mutational spectrum of de novo mutations in the offspring of *POLD1* L474P fathers to -PC1 showed a significantly higher value compared to the offspring of mothers with *POLD1* L474P, and to the offspring of wildtype parents (KS test *p*-value = 0.0019) (Fig. 2H, Supplementary Fig. S5).

Altogether, our analyses demonstrate that the presence of a mutator allele (in heterozygous state) could be more easily detectable from changes in the mutational spectra than from an increase in the overall mutation burden (Fig. 2F–H). More generally, deviation from the spectrum of wildtype trios can potentially be used to detect the presence of mutators in the population (Supplementary Note 3).

### Biallelic deficiency of Polδ proofreading is required for hypermutability

**Fig. 3:**
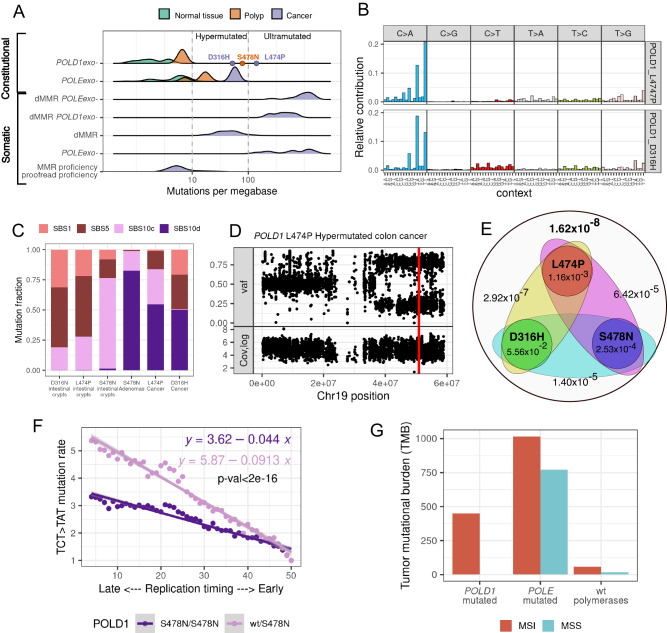
Biallelic inactivation of Polδ leads to hypermutability. **A** Top two rows: Mutability (mut/Mb) of normal and cancer colon samples from individuals with constitutional mutations in the exonuclease domain of *POLE* or *POLD1* (sequencing data obtained from Robinson et al. [6] and produced in this study). Dots mark hyper- and ultra-mutated samples from heterozygous carriers of constitutional pathogenic mutations in *POLD1* exonuclease (violet dots - two cancer samples sequenced in this study; orange dot – adenoma sample from Robinson et al. [6]). Bottom four rows: Mutation rates in endometrial cancer samples from The Cancer Genome Atlas (TCGA_UCEC) with somatic mismatch repair deficiency (dMMR) and/or polymerase proofreading deficiency (*POLE*exo- or *POLD1*exo-) (different combinations). **B** 96-nucleotide context mutational spectrum of the ultra- of hyper-mutated cancer samples from the *POLD1* L474P heterozygote (IV.1; Fig. 1A) and from a *POLD1* D316H heterozygote. **C** Fraction of mutations attributed to SBS10c and SBS10d mutational signatures in normal crypts, polyps^6^ and cancer samples from heterozygous carriers of *POLD1* variants. **D** Variant allele frequency (top) and total coverage (bottom) along chromosome 19 of the cancer samples of the *POLD1* L474P heterozygote (IV.1; Fig. 1A), showing cnLOH of the genomic region. The red line indicates the position of *POLD1* L474P. **E** Probabilities of cnLOH at one nucleotide site for each tumor and joint probabilities to observe cnLOH in two or three tumors simultaneously. **F** Fold change in mutation rate in bins of different replication timings compared to the bin of the earliest replication timing. The *p*-value corresponds to the significance of the interaction term between the replication timing bin and homozygosity status in a binomial regression model. **G** Mutation rate in TCGA_UCEC cancer samples with heterozygous somatic *POLD1* exonuclease mutations and varying statuses of MMR deficiency/proficiency. *Y*-axis represents the median of the distribution of tumor mutational burden in the corresponding subset of samples.

The proofreading function of human *POLE* and *POLD1* was previously suggested to be haploinsufficient [3–5]. However, unlike *POLE* exonuclease pathogenic variants, which strongly affect germline and somatic mutation rates even in a heterozygous state [6], we and others [6, 18] found only a minor effect of heterozygous *POLD1* L474P, and other *POLD1* pathogenic variants, on the mutation rate in non-tumoral tissues. To better understand how such a weak mutation rate modifier can drive a highly penetrant cancer phenotype [4, 5, 12, 19], we sequenced a tumor sample from the family with *POLD1* L474P (Fig. 1A; colorectal cancer developed by individual IV.1). In contrast to cultured fibroblasts, the sequenced tumor was characterized by an ultramutated phenotype, with 140 mutations per megabase (mut/Mb) (ultramutated tumors: >100 mut/Mb) (Fig. 3A). The mutational spectrum of the tumor also differed from the spectrum identified in the cultured fibroblasts of *POLD1* L474P heterozygous carriers and in the phenotypically normal crypts of carriers of different *POLD1* pathogenic variants studied by Robinson et al. [6] Specifically, the tumor sequence was enriched in C>A mutations (which comprised ~54% of observed mutations), particularly in TpCpA and TpCpT contexts (Fig. 3B). This mutational spectrum perfectly matched SBS10d (cosine similarity = 0.96), also found in hyper- or ultra-mutated polyps from carriers of the *POLD1* pathogenic variant S478N [6]. We also sequenced and analyzed a colorectal cancer developed by a carrier of the pathogenic variant *POLD1* D316H, which affects a catalytic site of the Polδ exonuclease. This tumor was also characterized by a high mutation rate (53 mut/Mb) and presence of SBS10d (Fig. 3C). These data show that the two *POLD1* exonuclease mutated cancers have an extremely high mutation rate and share the shift in mutational spectrum with a previously reported hypermutable adenoma obtained from an individual with constitutional *POLD1* S478N [6], suggesting a mutational process different from the mild mutagenesis observed in normal tissues of individuals with heterozygous *POLD1* pathogenic variants. The two analyzed tumors were microsatellite stable and had normal expression of the DNA mismatch repair (MMR) proteins MLH1, MSH2, MSH6 and PMS2, excluding inactivation of this DNA repair system. Of note, tumors with dysfunctional MMR and an error-prone version of Polδ usually have spectra that correspond to mutational signature SBS20 [20, 21], which was not observed here.

To explain the discrepancy between mutation rates and spectra of normal vs. tumor samples from heterozygous carriers of *POLD1* exonuclease pathogenic variants, we considered the possibility of somatic inactivation of the exonuclease function in the second copy of *POLD1* in the ultra- or hyper-mutated tumors. In fact, all three samples with elevated mutation rate, including the two tumors sequenced in this study obtained from heterozygous carriers of *POLD1* L474P and D316H, and the adenoma from a *POLD1* S478N heterozygote (sequence obtained from Robinson et al. [6]), had copy neutral loss of heterozygosity (cnLOH) of the *POLD1* region. In all three tumors, the cnLOH causes the loss of the wildtype *POLD1* allele, leading to homozygosity of the pathogenic *POLD1* exonuclease variant (Fig. 3D, Supplementary Fig. S6). Based on the overall number of structural variants identified in the three studied tumors, we estimated that the probability to observe cnLOH simultaneously in all three cases by chance was extremely low (p(cnLOH) = 1.62 × 10^−8^, p(LOH) = 9.92 × 10^−7^) (Fig. 3E, Supplementary Table S4), supporting the involvement of this rearrangement in the development of the hypermutable phenotype. Our findings suggest a high level of haplosufficiency of Polδ proofreading in human cells, showing that the presence of a single copy of wildtype *POLD1* can prevent a strong increase in mutation rate. Interestingly, we studied an MMR-proficient colon cancer (location: sigmoid colon) diagnosed in a 74-year-old patient with a constitutional *POLD1* D402N variant, who had undergone resection of 42 colorectal polyps. In this cancer sample, neither LOH, cnLOH, nor any somatic mutation affecting *POLD1* (or any *POLD1* subregion) could be identified in the second *POLD1* allele. Agreeing with our hypothesis, this tumor had a somatic mutation burden of ~8 mut/Mb, which is 10 times lower than in the three *POLD1*-associated hypermutable tumors described above, and comparable to the mutation rate observed in normal tissues of heterozygous carriers of *POLD1* exonuclease pathogenic variants. Moreover, the mutational spectrum of the tumor did not show a significant presence of SBS10d, but had a small contribution of SBS10c, specifically observed in normal tissues of heterozygous individuals (Supplementary Fig. S7). The age of cancer onset and the mutational features of the tumor suggest a sporadic origin for this tumor (phenocopy). These results further support the fact that heterozygous exonuclease deficiency of *POLD1* only mildly affects the mutation rate, and abrogation of the exonuclease proficient copy is required to promote hypermutability.

In line with our results, haplosufficiency of *POLD1* has been observed in yeast experiments [9]. A plausible explanation for this haplosufficiency is the ability of wildtype Polδ to proofread mismatches extrinsically, i.e., those produced by other mutant enzymes, thus preventing hypermutability. The abundance of TpCpA>A and TpCpT>A mutations in homozygous samples suggests that extrinsic Polδ proofreading is highly efficient for these types of mutations.

To identify and characterize the relative activity of the extrinsic proofreading effect of Polδ along the genome, we compared the relationship between replication timing and mutation rate in cells with homozygous and heterozygous *POLD1* L474P (our data) and S478N [6]. Both in hetero- and homozygous *POLD1*-mutated samples, mutation rates strongly depend on replication timing (Supplementary Fig. S8). However, in heterozygous samples this dependence is more pronounced for both S478N and L474P (Fig. 3F, Supplementary Fig. S9), suggesting that the extrinsic proofreading effect of *POLD1,* or its interaction with MMR, is stronger in early replicating regions.

## Discussion

Our findings indicate that constitutional heterozygous pathogenic variants in the exonuclease domain of *POLD1* have a mild effect on somatic and germline mutation rates in humans, although heterozygous carriers of *POLD1* exonuclease domain pathogenic variants are predisposed to develop hyper- or ultra-mutated cancers. We have observed, for the first time, that hypermutability in cancers or adenomas is associated with somatic loss of the wildtype *POLD1* allele. These results obtained in human cells and tumors agree with an extensive body of literature debating the recessive effect of *POLD1* exonuclease deficiency in yeasts and mice [7–11].

**Fig. 4:**
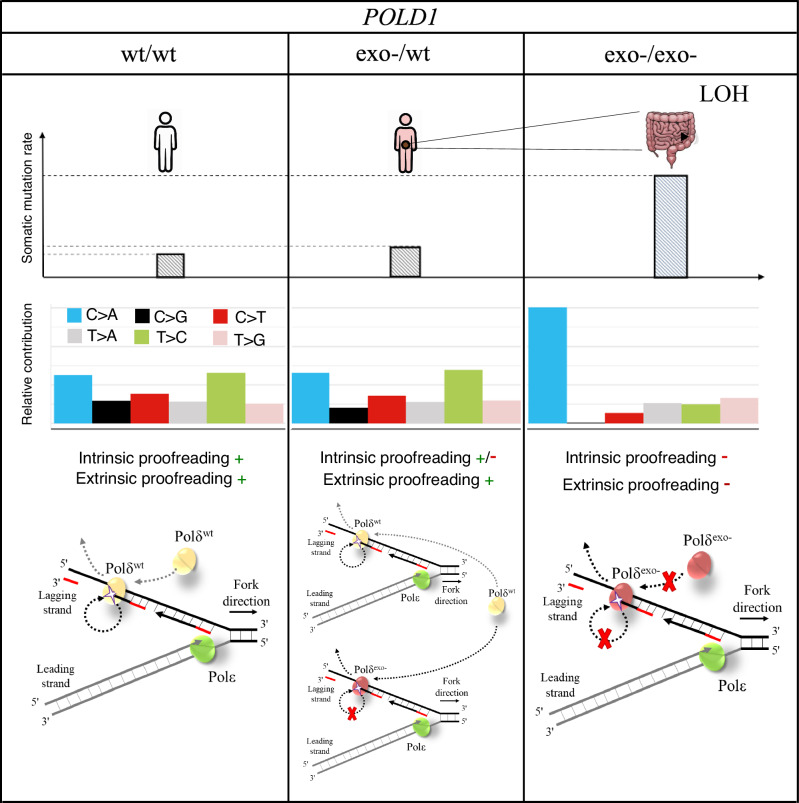
Schematic representation of the possible underlying mechanisms for monoallelic and biallelic inactivation of *POLD1* proofreading activity.

As in the case of *POLE*, it would be intuitive to expect that *POLD1* exonuclease deficiency had an additive effect on mutation rate, because mutated and non-mutated *Polδ* likely synthesize comparable amounts of DNA during replication of the lagging strand. Based on the results obtained in yeast and mice, which otherwise suggested a recessive behavior for *POLD1* exonuclease mutations, different hypothesis had been proposed, including a higher processivity of wildtype Polδ compared to the mutant allele [7], compensation by MMR [10], or prevalent expression of the wildtype copy of *POLD1* [22] among others. A recent study in yeast showed that the wildtype Polδ can correct mismatches produced by the mutant copy (extrinsic proofreading activity) [9]. Our data in human cells are consistent with this explanation, and thus, we hypothesize that Polδ extrinsic proofreading helps prevent hypermutability in cells with heterozygous *POLD1* exonuclease pathogenic variants (Fig. 4).

Our results are well suited to explain the prevalence of different DNA repair deficiencies in cancer, where a high mutation rate is a selected phenotype during cancer development. Here, we found that heterozygous mutations in the exonuclease domain of *POLD1* lead to just a minor increase in mutation rate if other DNA repair systems are intact, suggesting that heterozygous *POLD1* mutations should be almost neutral on a normal genetic background. In fact, to our knowledge, there are no reported tumors where *POLD1* is somatically mutated, but MMR is intact. Meanwhile, somatic inactivation of the second copy of *POLD1* on the background of a heterozygous constitutional *POLD1* exonuclease mutation is under strong positive selection and is likely a major avenue for cancer development. We could expect somatic MMR inactivation as an alternative mechanism; however, dMMR is rarely found in cancers from individuals with constitutional heterozygous *POLD1* mutations (Supplementary Table S5). This probably reflects the higher odds to mutate the second copy of *POLD1* compared to achieving dMMR.

Interestingly, tumors with somatic *POLD1* exonuclease mutations seem to be always accompanied by dMMR. In these tumors, the results obtained for mutational signature analysis suggest that dMMR precedes *POLD1* proofreading deficiency (Supplementary Fig. S10). MMR deficiency alone increases the mutation rate, represents an advantageous genotype and could be fixed during tumor development; however, after a clonal expansion of dMMR, *POLD1* exonuclease mutations will further increase the mutation rate by an order of magnitude (Fig. 3G) and thus, should also cause positive selection. These observations may explain why only a heterozygous *POLD1* exonuclease mutation is rarely sufficient to promote transformation; either mutation (LOH) of the second *POLD1* allele or dMMR [23] must occur for fixation of a somatic mutation affecting *Polδ* exonuclease domain.

Heterozygous carriers of constitutional pathogenic variants in the exonuclease domain of *POLD1* are mainly predisposed to multiple adenomatous gastrointestinal polyps, as well as colorectal and endometrial cancers, among other tumor types, which largely overlap the phenotypic features of other autosomal dominant colorectal cancer predisposition syndromes associated with defects in DNA repair components, such as those caused by *POLE* proofreading deficiency or dMMR (Lynch syndrome). This tissue specificity may be explained by the fact that highly regenerative/proliferative tissues, such as the colon mucosa or the endometrial epithelium, are more sensitive to the deficiency in DNA repair mechanisms that deal with replication errors (polymerase proofreading defects, mismatches at the DNA replication forks, etc.), because inactivation of these systems will lead to extensive mutation accumulation [24]. Another hypothesis for this tissue specificity might involve higher LOH levels in those epithelia. However, the fact that recessive polyposis and cancer syndromes caused by a deficiency in other DNA repair mechanisms, e.g. those caused by constitutional biallelic mutations in *NTHL1*, *MUTYH* and *MBD4*, also target the colorectal epithelium (and some predispose to endometrial cancer too), argues against it.

In summary, in this study, we showed that heterozygous constitutional *POLD1* L474P has a minor effect on mutation burden in germline and soma but leads to a prominent change in mutational spectra. Sequencing a large number of trios with mutations in genes involved in replication could help better understand the mutational mechanisms in the germline and identify the relative roles of replicative errors and DNA damage.

We also uncovered a recessive effect of *Polδ* proofreading deficiency in the context of cancer, suggesting that a constitutional heterozygous *POLD1* exonuclease mutation may lead to cancer through a double hit mechanism, similarly to the somatic inactivation of the second MMR gene copy in Lynch syndrome patients [25–27].

This and other findings suggest that the non-additive effect of polymerase proofreading and DNA repair genes inactivation on mutation rate is translated into recessivity and/or epistatic interactions in cancer. Also, implementation of tumor findings, i.e., presence of a somatic second hit in *POLD1* together with the detection of mutational signature SBS10d, into *POLD1* variant classification approaches will help interpret the functional effect of the variants identified in the clinic and better characterize the PPAP tumor spectrum.

### Supplementary information

Supplementary Material

## Data Availability

DNA sequencing data are deposited in the European Genome-Phenome Archive (EGA) with accession code EGAS00001006434. Called de novo and somatic mutations are available online (https://figshare.com/account/home#/projects/156404).

## Code availability

Code required to reproduce the analyses in this article is available online (https://github.com/andrianovam/POLD1-project_scripts).
