# Supplementary material for: Discovery of recessive effect of human polymerase δ proofreading deficiency through mutational analysis of POLD1-mutated normal and cancer cells
Source: Eur J Hum Genet. 2024 Apr 24;32(7):837–45. doi: 10.1038/s41431-024-01598-8 (PMC11219999; doi:10.1038/s41431-024-01598-8)
Supplement: Supplementary file 1 — Supplementary Material [file 41431_2024_1598_MOESM1_ESM.pdf]

Supplementary Material for

Discovery of recessive effect of human polymerase δ proofreading deficiency through mutational analysis of POLD1-mutated normal and cancer cells

Maria A. Andrianova†, Vladimir B. Seplyarskiy†, Mariona Terradas, Ana Beatriz Sánchez-Heras, Pilar Mur, José Luis Soto, Gemma Aiza, Emma Borràs, Fyodor A. Kondrashov, Alexey S. Kondrashov, Georgii A. Bazykin, Laura Valle*

†These authors contributed equally

*Correspondence to: LV (lvalle@idibell.cat)

Table of contents

Supplementary Material and Methods

Supplementary Note 1

Supplementary Note 2

Supplementary Note 3

Supplementary Figure S1.

Supplementary Figure S2.

Supplementary Figure S3.

Supplementary Figure S4.

Supplementary Figure S5.

Supplementary Figure S6.

Supplementary Figure S7.

Supplementary Figure S8.

Supplementary Figure S9.

Supplementary Figure S10.

Supplementary Figure S11.

Supplementary Figure S12.

Supplementary Figure S13.

Supplementary Figure S14.

Supplementary Figure S15.

Supplementary Figure S16.

Supplementary Figure S17.

Supplementary Figure S18.

Supplementary Figure S19.

Supplementary Table S1.

Supplementary Table S2.

Supplementary Table S3.

Supplementary Table S4.

Supplementary Table S5.

Supplementary References

SUPPLEMENTARY MATERIAL AND METHODS

Study participants and Ethical approval

The family included in the study was recruited through the Hereditary Cancer Genetic Counseling and Molecular Genetics Lab at the University Hospital of Elche (Spain), where the clinical information and blood and skin punches were obtained. FFPE tumor material was obtained through the Valencian Biobank Network.

Experimental procedure to assess somatic mutation accumulation in fibroblasts

Fibroblast obtention and immortalization

A skin punch biopsy was obtained from individuals III.2, III.4, IV.1, IV.2, IV.3, IV.4, IV.5, and IV.6 (Fig. 1; Suppl. Table S1). The obtained biopsies were kept on culture media [DMEM with 10% fetal bovine serum and 100U/ml penicillin-streptomycin (Gibco, Thermo Fisher Scientific, Walthman, MA] at 4ºC until processing. After a 1X PBS wash, the sample was incubated ON at 37ºC with digestion media (DMEM supplemented with 160 U/ml collagenase and 1.25 U/ml dispase) and then disaggregated by pipetting. The sample was washed with culture media and seeded in one well of a 12-multiwell plate and maintained at 37ºC in a 5% CO_2 atmosphere. Cells were expanded for two passages before immortalization with lentiviral transduction with hTERT.

The lentiviral plasmid carrying the catalytic subunit of human telomerase protein (pLVX-IRES-hTERT-tdTOMATO) as well as envelope and packaging plasmids (pPAX2 and pMD2G) were kindly provided by Dr. Manel Esteller. Lentiviruses were produced transfecting HEK293 cells growing on a T75 flask with 20ug of total DNA and Lipofectamine 2000 (Thermo Fisher Scientific) for 16h. The culture medium was changed, collected at 72h and filtered using a 0.45 µm filter. The lentivirus enriched-medium was immediately used to transduce the human fibroblasts growing on a T25 flask in the presence of 8µg/ml polybrene (Sigma-Aldrich, San Luis, MO). Infected fibroblasts were maintained in culture until having enough cells for cell sorting. Cells expressing hTERT-tdTOMATO were enriched by fluorescent activated cell sorting (FACS). Cells were analyzed with the cell sorter MoFlo Astrios (Beckamn Coulter, Brea, CA) using a 561nm laser. Immortalized fibroblasts were maintained in culture media at 37ºC in a 5% CO_2 atmosphere and split at 1:3 ratio.

Single cell isolation and clonal expansion

To generate single-cell clones, fibroblasts enriched in hTERT-tdTOMATO were single-cell sorted using the MoFlo Astrios and the 561 nm laser. Each single cell was automatically plated

in a well of a 96-well plate in the presence of 200 µl of culture medium. The individual cultures were followed up to ensure clonal expansion. Each pool of cells was passed to growing sizes of culture plates up to T25 flasks. The first confluent T25 flask was considered the starting passage (p0). Two clones per individual were maintained in culture for 30-45 additional passages (Suppl. Table S2) as described above. One clone per individual was sequenced.

DNA extractions

Peripheral blood DNA was extracted using the FlexiGene DNA kit (Qiagen, Valencia, CA). DNA from fibroblasts was obtained with the Quick-DNA Miniprep Plus kit (Zymo Research, Orange, CA). DNA from buccal swabs obtained with Isohelix swab packs and maintained in BuccalFix tubes (Isohelix, Cell Projects Ltd, UK) was extracted using a standard phenol-chloroform protocol. DNA from formalin-fixed paraffin-embedded (FFPE) tumor samples was isolated with the kit QIAamp DNA FFPE tissue Kit (Qiagen). All extractions were carried out following the manufacturers' instructions.

Whole-genome sequencing (WGS)

DNA preparation for WGS was performed with the TruSeq Nano DNA Library, and sequencing was carried out in a NovaSeq 6000 150 PE (2x150 bp). Sequencing was performed at a minimum coverage of 90 Gb (30x) for the germline studies in the family members, and at a minimum coverage of 150 Gb (50x) for the experiments with fibroblasts (P0 – ~P40 passages of the fibroblasts' cultures). Sequencing was performed at Macrogen (Macrogen Inc, Seoul, South Korea).

Whole-exome sequencing

Exome sequencing was performed in FFPE tumor DNA of individual IV.6. Exome capture was performed with Kappa HyperExome Probes (Roche) and sequenced in a NovaSeq 6000 S1 (2x100bp). Sequencing was performed at Centro Nacional de Análisis Genómico (CNAG, Barcelona, Spain).

Analysis of MMR status in tumors

MMR status in tumor tissue was assessed by standard methods, using immunohistochemistry of MMR proteins MLH1, MSH2, MSH6 or PMS2, and/or by analysis of microsatellite instability (MSI) by PCR-based analysis of microsatellite markers.

Variant calling in fibroblasts and post-processing filters

Sequenced reads were aligned to hg19 reference human genome downloaded from UCSC (https://hgdownload.soe.ucsc.edu/downloads.html#human) using Burrows–Wheeler alignment (BWA-MEM) (1).

Somatic mutations in single cell-derived colonies were called using Mutect2. DNA genome sequencing data available from normal tissue (blood, buccal swab, or fibroblasts) from the corresponding individual was used as matched normal DNA. Panel of normals (--panel-of-normals Mutect2 argument) was created from all available sequenced blood samples. The population allele frequencies in gnomAD were used as a prior for germline variant detection (--germline-resource Mutect2 argument). Additional filter for the variant allele frequency observed in sequenced data was applied: only mutations with variant allele frequencies (VAFs) between 0.25 and 0.75 were selected for the analysis.

Somatic mutations accumulated during the experiment were called using Mutect2. Mutations present in the end point (P40) but absent in the start (P0) were selected. To filter out recurrent technical artifacts, all available sequencing data from samples that did not correspond to the mutation accumulation experiment were used to create a panel of normals (--panel-of-normals Mutect2 argument). The population allele frequencies in gnomAD were used as a prior for germline variant detection (--germline-resource Mutect2 argument).

Mutational spectra and PCA analysis

The 96-mutational spectrum was calculated dividing the number of mutations of a particular type in fixed 3-nucleotide contexts by the total number of mutations of that type. Principal component analysis (PCA) was used on these 96-dimensional vectors for mutations obtained from fibroblast colonies. Mutational spectra obtained by the same procedure across different datasets (Fig. 2F, Fig. 3C, E, Fig. 3G, H) were projected onto the obtained PC space.

Extraction of *de novo* signatures

Mutational signatures in fibroblasts were extracted *de novo* and then decomposed to COSMIC signatures using SigProfilerExtractor (2). The number of mutations attributed to each COSMIC signature was obtained as output.

For mutations accumulated in fibroblast colonies during the experiment, SigProfilerExtractor was run with the following parameters: minimum_signatures=1, maximum_signatures=15, nmf_replicates=300. The solution with the most stable signatures (n_signatures = 2) was selected. Two de novo extracted signatures were decomposed in 6 reference COSMIC signatures. The initial mutational pattern of each sample was refitted to COSMIC signatures

using the SigFit package. To avoid overfitting the subset of signatures for decomposition, the analysis was limited to the six signatures predicted by SigProfilerExtractor: SBS5, SBS10c, SBS36, SBS37, SBS45 and SBS93.

The 'sigfit' R package (3) was used to estimate exposure to mutation signatures in intestinal crypts, adenomas and cancer samples with constitutional *POLD1* mutations. In this case, the signatures fitted were limited to SBS1, SBS5, SBS10c and SBS10d. The 'mSigAct' package was used to estimate the significance of SBS10c presence (4).

Analysis of insertions and deletions

Insertions and deletions were called in the same procedure described in "Variant calling in fibroblasts and post-processing filters". Following analyses were performed using "MutationalPattern" package in R (5). Refitting was made with "strict" method and max_delta=0.004.

Calling of germline de novo mutations and post-processing filters

Sequenced reads were aligned to the hg19 reference human genome downloaded from UCSC using Burrows–Wheeler alignment (BWA-MEM). *De novo* mutations were called using standard GATK4 best practices pipeline. All samples were jointly called, but only high confidence mendelian violation sites with minimum GQ=20 for each trio member were selected. Subsequently, additional filters were applied: i) coverage of each trio member ≥10; ii) absence of reads confirming alternative allele in parents; iii) number of reads confirming reference and alternative allele in proband ≥5; iv) allele frequency of the alternative allele in the proband ≥0.3. Additionally, clustered mutations (distance between mutations ≤1000bp) were filtered out as potential false positives. Mutations present in any other family member were also excluded. To eliminate false positive mutations coming from sequencing of proband fibroblasts, we used other fibroblasts from the same individual (used in MA experiments) as additional confirmation of mutation presence: true *de novo* mutations must be present in all tissues of the proband, including the other fibroblast sample. This additional filter mainly removed variants from the left tail of the variant allele frequency distribution of potential *de novo* mutations, thus keeping the distribution more symmetric around 0.5 (Suppl. Fig. S11). To evaluate the performance of the filter, we took the data from our preliminary sequencing of blood samples for two trios from the family (IV.5 and IV.6 probands) and compared the *de novo* mutations obtained in the two attempts. We found out that for the IV.6 offspring, this additional filter removed 1257 out of 1360 variants. Only 6 of them had been proposed as *de*

novo mutations in the first round of trio sequencing. For the IV.5 individual, the filter removed 56 out of 121 candidates and none of them had been called as *de novo* mutations in the preliminary sequencing (Suppl. Fig. S12). Blood samples for each individual in the trios had been preliminary sequenced with 20X coverage, *de novo* mutations had been called using PhaseByTransmission GATK3 tool (--prior = 1e-4) and sites with violation from mendelian inheritance were selected.

Enrichment of *de novo* mutations in *POLD1* contexts

According to analysis of mutations in fibroblasts, four contexts were enriched in mutations in carriers of *POLD1* L474P variant: CpCpT>A, TpCpT>A, ApTpT>A and CpTpT>G.

The calculated proportion of mutations in these contexts in *de novo* mutations of published trios equalled 2.4%. This is thus the expected proportion for such mutations in offspring of wildtype *POLD1* parents. We then calculated the expected number of such mutations in each offspring in our experiment by multiplying the expected proportion by the total number of observed mutations in offspring. The sum of expected numbers calculated by all offspring of father-carriers of *POLD1* L474P variant was compared to the observed sum to estimate the enrichment. Rate-ratio test was used for comparison of observed vs. expected data.

The proportion of mutations in *POLD1* contexts in somatic cells was calculated using mutations from *POLD1* L474P fibroblasts excluding IV.6 and IV.1 samples.

Simulation of *de novo* mutations and test for the presence of mutators in the population

To test how many offspring of wildtype parents in the sample have –PC1 values higher than the mean value of –PC1 in the offspring of fathers harboring *POLD1* L474P by chance, we generated an artificial dataset of trios by randomly sampling *de novo* mutations according to their fractions in homogeneous underlying spectra. To obtain the underlying spectra, we used data from 6233 offspring from two publicly available datasets, aggregated all *de novo* mutations together and calculated the proportion of each mutation type in each possible 3-nucleotide context. For each offspring in the dataset, we counted the observed number of *de novo* mutations, and sampled the same number of mutations from the obtained spectrum. For each simulated set of *de novo* mutations, we created the vector of mutational probabilities and estimated the value of the -PC1 component by projecting in the PC space obtained previously from the analysis of fibroblasts. We repeated the same procedure generating two or more offspring for each family and averaging the –PC1 among siblings.

Similarly, a sample of trios with a mixture of offspring of *POLD1* L474P carriers was simulated. For 95% of the offspring, we sampled mutations according to their probability in the wildtype trios and for the remaining 5% of samples, we sampled mutations based on their probability of occurring in the offspring of *POLD1* L474P fathers in the studied family. We then calculated the mutational spectrum per simulated individual and the -PC1 values.

For other mutagenic processes (Polε proofreading inactivation, defective base excision repair) *de novo* mutations for mutators (proportion of mutators varied from 0 to 10%) were sampled as a mixture of mutations from wildtype trios spectrum and spectrum of the corresponding known COSMIC signature attributed to the mutagenic process (SBS10a, SBS36 correspondingly). Cosine similarity with the COSMIC signature was used to estimate the presence of this signature in the simulated spectrum of the sample. Kolmogorov-Smirnov test was used to estimate the significance of difference between simulated subsets: subset of wt trios vs subset with mutators (Suppl. Fig. S13).

For simulation in Suppl. Fig. S4 we created a synthetic dataset of *de novo* mutations for 5000 wild type trios as a background. The number of mutations per individual was generated from negative binomial distribution (mu=60, size=80) and mutations were sampled from the observed spectrum of *de novo* mutations in wt trios. For 5 samples in addition to this procedure we added mutations sampled from SBS10c signature spectrum. The number of additional mutations was generated from the Poisson distribution with lambda equal to 0.15 multiplied by the number of mutations from the wt spectrum. Projection to PC1 and proportion of *POLD1*-specific contexts were used to estimate presence of mutations introduced by additional mutagenic process (Polδ proofreading inactivation).

Calling of mutations in tumors from *POLD1* pathogenic variant carriers

Sequenced reads were aligned to the hg19 reference human genome downloaded from UCSC (https://hgdownload.soe.ucsc.edu/downloads.html#human) using Burrows-Wheeler alignment (BWA-MEM). Somatic mutations in tumor sample sequenced in this study were called against normal sample from the same individual using standard GATK4 best practices pipeline. The Learn Orientation Bias Artifacts tool was used to control for orientation bias, which is critically important for FFPE samples. Mutations with variant allele frequency <0.15 were filtered out, as low-frequency variants are known to be enriched in formalin fixation artifacts in FFPE samples (6).

Two additional analyzed cancer samples (*POLD1* D316H and D402N) didn't have matched

normal samples from the same individuals. Mutations there were also called using standard GATK4 best practices pipeline with Mutect2 in "tumor-only mode" and with The Learn Orientation Bias Artifacts tool. Variants present in less than 20 reads, with variant allele frequency <15% (potential artifacts) and variants with vaf higher than 0.4 (potential remaining germline) were excluded from the subsequent analyses.

Epigenetic covariates for the extrinsic proofreading effect of Polδ

Genome was splitted in 100-kb non-overlapping windows. The number of mutations and target sites was calculated in each window. The replication timing (RT) is highly conserved between human tissues and cell types (7, 8). Here we used the mean replication timing for each window obtained using Wavelet-smoothed Signal for Repli-seq data for HeLa-S3 cell line (GSE34399). Windows were splitted in bins according to RT mean value; mutation rate in each bin was calculated as a sum of all mutations in windows in this bin divided by the sum of target sites. The obtained mutation rate in each bin was normalized for the mutation rate in the bin of the earliest replication time.

For S478N samples whole genome data was available. For heterozygous S478N, we used data from 32 samples from 6 individuals (48052 mutations in total). For homozygous S478N we were able to use only 1 sample from 1 individual with a total of 66774 mutations. Mutational data were obtained from a previously published dataset (9). Only TpCpT>A/ApGpA>T mutations were analyzed. and 50 RT bins were used. To estimate statistical significance of the difference, we ran binomial regression with interaction using RT bin and homozygosity of S478N variant as predictors. The p-value for the interaction term is provided in Fig 4E.

For L474P samples whole exome data was available, all mutations were analyzed together, and RT was split in 3 bins only.

LOH rate estimation

We called LOH regions in all three samples independently using CNVkit (10), and calculated the rate of LOH in each sample dividing the number of sites with LOH by the total number of called target sites. To get an unbiased estimation, we excluded chr19 from this estimation as we wanted to check the independence of LOH in the *POLD1* gene located in that chromosome. The probability to observe LOH in all three samples was obtained as the product of three probabilities.

SUPPLEMENTARY NOTE 1

Evidence that supports the pathogenicity of *POLD1* L474P and its influence on the proofreading activity of Polδ. The ACMG/AMP guidelines adapted to the *POLD1* gene (11, 12) classifies L474P as likely pathogenic (evidence and rule codes highlighted in blue, between brackets).

- Germline carriers show the phenotypic characteristics of the hereditary cancer syndrome associated with polymerase proofreading deficiency (PPAP). The variant segregates with the cancer and/or polyposis phenotypes (13-16). [PP1_strong: when co-segregation is observed in ≥7 meioses in ≥2 families (reported L474P families: 9 meiosis in 4 families)]
- Absence in population databases (zero individuals in gnomAD v.2 containing 125,748 exomes and 15,708 genomes, or in gnomAD v.3 containing 76,156 genomes) [PM2_supporting: L474P is absent in control populations]
- It affects a highly conserved residue within the Exo IV motif of the exonuclease domain of Polδ. The variant is predicted pathogenic by computational tools (REVEL (metapredictor) score = 0.913; "1" being the highest possible value for pathogenicity (17). [PP3: Missense ED variants with a REVEL score ≥0.5]
- Modification of the L474 residue leads to proofreading dysfunction (experiment performed in yeast (*Saccharomyces cerevisiae*) (18)). Own experimental data in *Schizosaccharomyces Pombe* show an effect of L474P on the proofreading activity (hypermutator phenotype) (Suppl. Fig. S1). [PS3_supporting: hypermutator phenotype observed in a yeast-based experimental system]

SUPPLEMENTARY NOTE 2

Mutations accumulated during life

Similar to a previous study carried out by Robinson et al. (9), we searched for the footprint of polymerase delta (Polδ) proofreading deficiency in mutations accumulated in tissues over the lifetimes of the studied individuals. For this, we analyzed the mutational patterns obtained from the genome sequencing of the single cell-derived fibroblast colonies of eight family members (six *POLD1* L474P heterozygotes and two *POLD1* wildtype individuals; Fig. 1A, blue rectangles). Overall, the number of observed mutations correlated with the individual age at the time of biopsy (Suppl. Fig S14). Among the mutations accumulated in the skin fibroblasts of the individuals over their lifetime high fraction of observed mutations were caused by ultraviolet (UV) irradiation: on average, approximately 9,260 SNVs (~52% of the total number of SNVs) were attributed to mutational signature SBS7 and a high proportion represented by CC>TT double substitutions (Suppl. Fig S15). Of note, the number of identified UV-induced mutations was higher than previously reported for skin fibroblasts (19) and more in line with the numbers reported for melanocytes (20).

We observed the SBS10c signature, corresponding to Polδ proofreading deficiency, in all carriers of L474P. Unexpectedly, mutations attributed to SBS10c were also identified in non-carrier family members, although in much smaller numbers. A plausible explanation for this observation could be a misattribution due to the refitting procedure applied to a fixed subset of signatures (see Suppl. Materials and Methods). In fact, simulations confirm that imperfection of signature decomposition could identify spurious signatures (Suppl. Fig S16). An orthogonal approach using PCA also confirms the presence of the Polδ proofreading deficiency signature (increased values of -PC1) in carriers of L474P (Fig 2D). The separation from non-carriers was however fuzzier compared to the experimental data, in line with the results of signature analysis, and suggesting a noisier structure of the data.

SUPPLEMENTARY NOTE 3

Detection of mutators in the population based on deviation from the spectrum of wildtype trios

Small number of de novo mutations per individual makes it difficult to identify mutators with the small effect on mutation rate, as in the case of *POLD1* L474P. In this case the increase in the number of mutations could be masked by the variance of Poisson distribution of the number of mutations. However, if the mutator has very biased preferences in the mutational context, its presence could be identified with higher power from the deviation of the mutational spectrum from that of the wildtype trios (Fig 2F-H, Suppl. Fig S5). If an additive model of mutational processes is assumed, this observation might seem counterintuitive. However, even in that scenario, a small number of mutations originating from an additional mutagenic process may result in a shift in the mutational spectrum if the spectrum of the additional mutational process strongly differs from the background processes, even though the absolute number of those mutations can be low (simulations supporting this hypothesis are shown in Suppl. Fig. S13).

Recently, pathogenic variants in known DNA repair genes were shown to contribute to germline hypermutation in sequenced human trios (21). We wondered if the offspring of parents with deficient Polδ proofreading activity contribute to the public datasets of sequenced families. To address this question, we mapped onto our PCA coordinates the mutational spectra of 6,233 offspring belonging to 4,638 families with assumedly wildtype *POLD1* status. Although the mean value of -PC1 in this sample was significantly lower than that in the offspring of *POLD1* L474P fathers, we found that 138 (2.2%) of the offspring had –PC1 above this value (Fig. 2H). The observed proportion of samples with a high –PC1 value could indicate the presence of undetected Polδ deficiency in parents of these trios; alternatively, it could reflect stochasticity in low count data. To distinguish between these alternatives, we generated an artificial dataset of trios by randomly sampling mutations according to their fractions in the spectra of *de novo* mutations in offspring of wildtype parents. Among these simulated trios, 2.0% of the offspring had values of -PC1 above the mean value observed in the five offspring of the two *POLD1* L474P fathers studied here (Suppl. Fig S17A). The presence of such cases in our simulated dataset indicates that a high value of -PC1 in some of the trios could be obtained by chance, and a comparable proportion of such cases in the data and in the simulation suggests that either *POLD1* mutated fathers are absent in the dataset or their fraction is smaller than the resolution of this method. To estimate the fraction of fathers with *POLD1* L474P that would detectably change the distribution of -PC1 values in a dataset, we generated an additional synthetic cohort

by mixing simulated offspring with mutations sampled from the *de novo* spectrum of wildtype parents with small fractions of simulated offspring with mutations sampled from the spectra of offspring of *POLD1* L474P fathers. For the 1% admixture, the proportion of samples with -PC1 above the mean observed in the offspring of *POLD1* L474P fathers was 2.5%, similar to the simulation with wildtype parents only. For the 5% admixture, the distribution had a pronounced shift to higher -PC1 values, and the corresponding proportion was 3.98% (a 1.8-fold increase compared to the wildtype simulation) (Suppl. Fig. S17B). We can thus conclude that the presence of a mutation rate modifier is detectable in a sample of trios based on the heterogeneity in the mutational spectrum in the offspring if the fraction of parents carrying the modifying mutation is substantial (e.g. ~5% for *POLD1* L474P).

Sequencing more than one child in a family should provide more power to detect families with deficiency in Polδ proofreading, as it is unlikely to obtain high values of -PC1 in multiple offspring by chance alone. By simulating multiple offspring per family with wildtype parents, we estimated that only 0.16% of the quartets with two sequenced siblings, and 0.04% of the quintets with three sequenced siblings, are expected to have the -PC1 value averaged between siblings higher than the mean -PC1 value in the offspring of fathers harboring *POLD1* L474P (Suppl. Fig S18). This confirms that sequencing more than one child per family decreases the rate of false positive prediction of a mutation rate modifier presence in the father, thus increasing the power of this approach. Among the 4,638 analyzed families, 1,555 were quartets with two sequenced siblings. Among them, the observed fraction of families with increased -PC1 value averaged between siblings (0.19%) was similar to the 0.16% estimated from the simulation, again arguing against a detectable contribution of families with Polδ deficiency to the studied dataset.

As mentioned above, our results indicate that the presence of an additional mutagenic process has more effect on the mutational spectrum of *de novo* mutations than the overall number of mutations (Suppl. Fig S5), and deviation from the spectrum of wildtype trios can potentially be used to detect a presence of mutators in the population. This observation can be generalized to any germline mutagenic process. Application of our method to different repair pathways characterized by unique spectra in synthetic datasets suggested that only processes affecting at least 3-7% of a population could be thus detected (Suppl. Fig S19). Such high frequencies of mutators for rare and highly severe varions, such as Polδ or Polε proofreading deficiency, in natural populations seem unrealistic. Potential application of this method to more common mutagenic processes needs further investigation.

SUPPLEMENTARY FIGURES

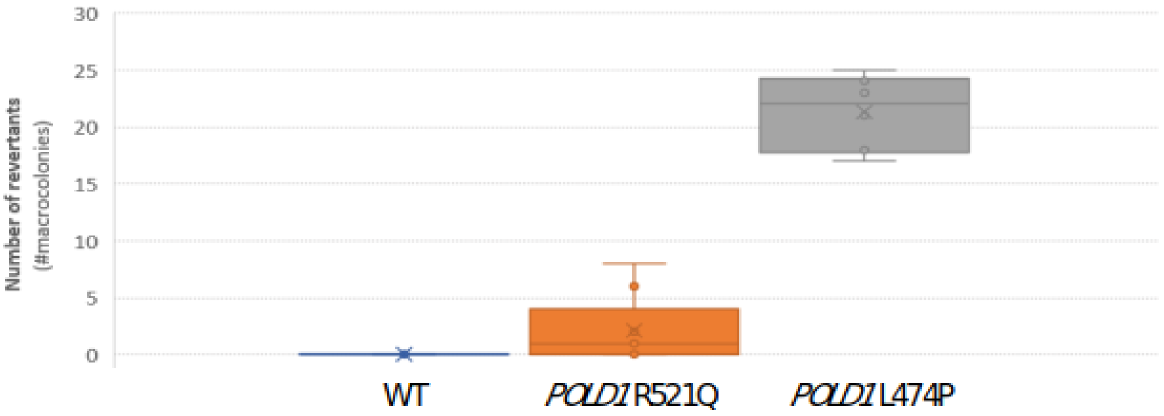

Supplementary Figure S1. Number of revertant colonies (per plate) in different genotype backgrounds (wildtype, *POLD1* R521Q and *POLD1* L474P) in *Schizosaccharomyces pombe* (haploid strain). Colony growth occurs when spontaneous mutations cause the reversion of the ade6-485 allele in the corresponding yeast strain due to polymerase proofreading deficiency. No growth of revertant colonies is expected in the WT strain. The mean and standard deviation was obtained from two independent experiments performed in triplicate. The three variants were assayed in parallel. The methodology used was described previously (22). *POLD1* variant nomenclature in the figure corresponds to the human gene. R521Q is a variant of unknown significance in the exonuclease domain of *POLD1*.

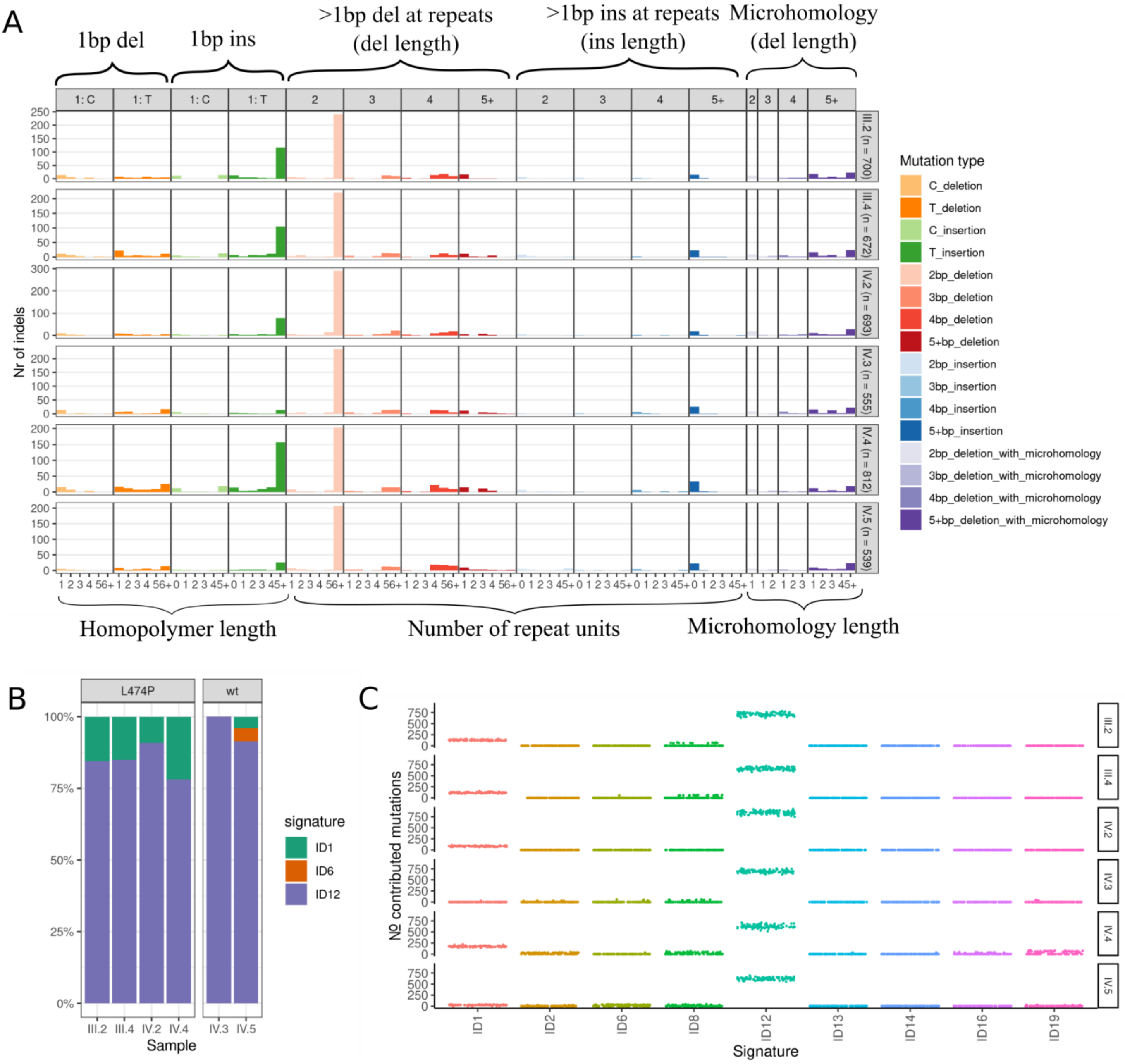

Supplementary Figure S2. Insertions and deletions accumulated during the experiment in fibroblasts colonies with heterozygous *POLD1* L474P. **A)** Mutational profile of accumulated insertions and deletions. Values in brackets near the sample names correspond to overall observed number of insertions and deletion. **B)** Decomposition of the observed spectra to COSMIC IDs signatures. **C)** Stability of the decomposition results in 100 bootstraps.

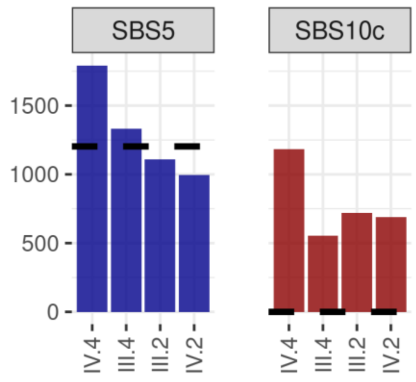

Supplementary Figure S3. Excess of mutations corresponding to the different mutational signatures in the cultured fibroblasts of *POLD1* L474P heterozygous carriers. The black dashed lines mark the mean number of mutations attributable to the corresponding signature in wildtype fibroblasts.

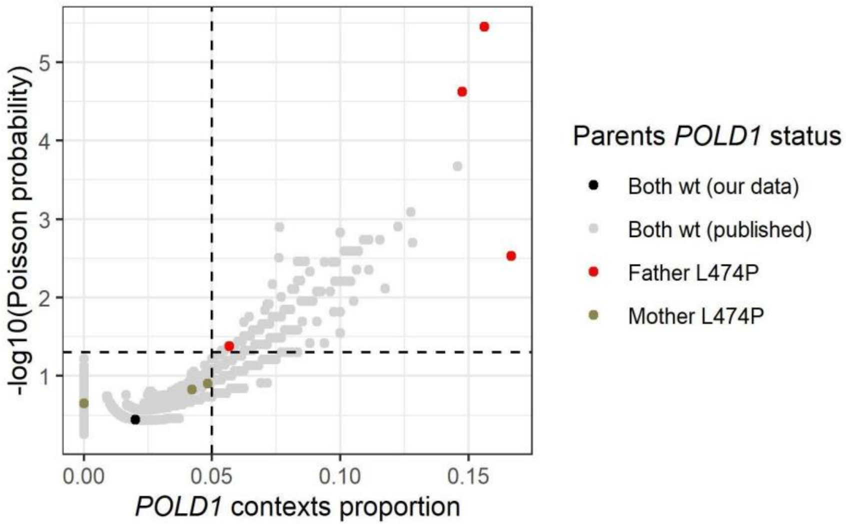

Supplementary Figure S4. Proportion of mutations in four 3-nucleotide contexts specific for mutated *POLD1* against Poisson probability of observed number of mutations in these contexts. Dashed horizontal line marks p-value=0.05. Dashed vertical line marks 90%-percentile of contexts proportion in published trios (23, 24).

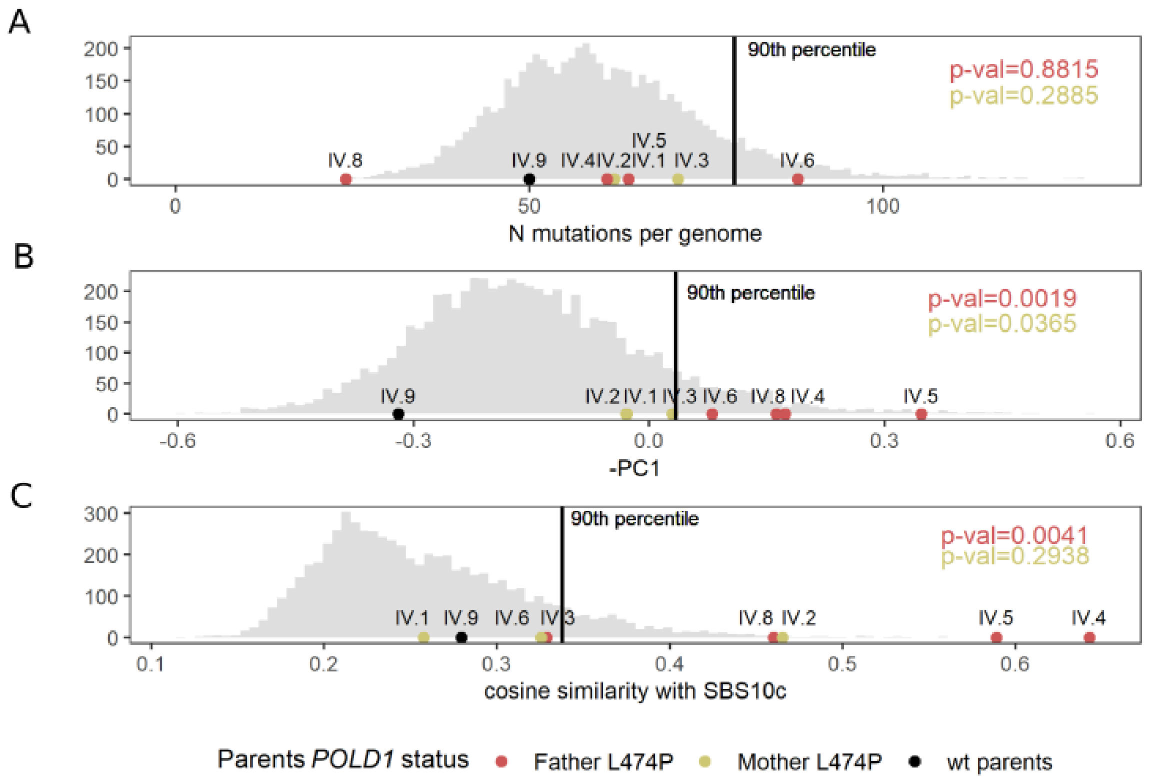

Supplementary Figure S5. Representation of the number of mutations (**A**), -PC1 values (**B**), and cosine similarity with SBS10c signature (**C**) in sequenced trios from the family compared to the distribution of the corresponding parameter in publicly available trios (23, 24). The p-value for Kolmogorov-Smirnov test of public trios against fathers-carriers of *POLD1* variant is shown in red, and against mothers-carriers of *POLD1* variant is shown in khaki.

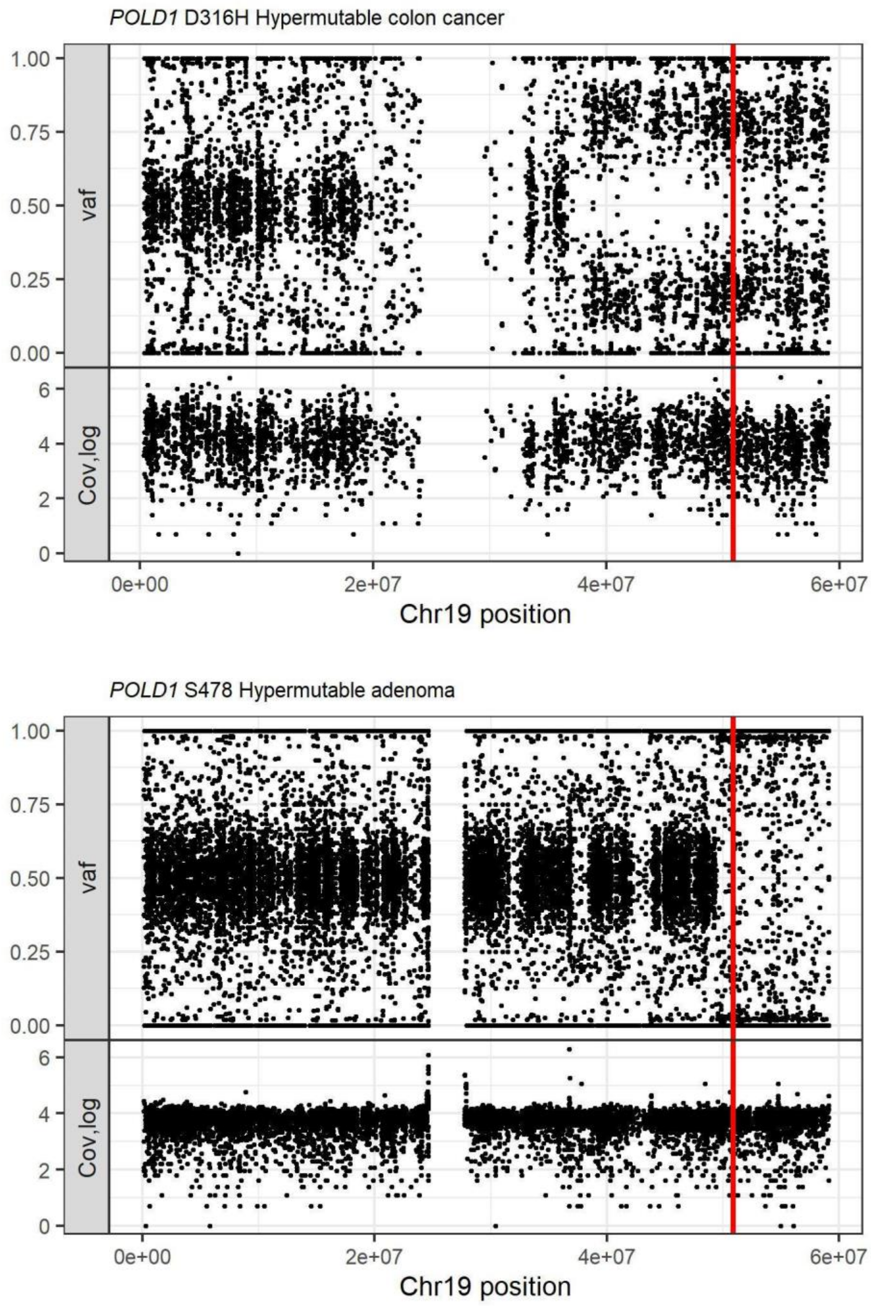

Supplementary Figure S6. cnLOH in tumor from germline carrier of *POLD1* D316H and in a hypermutable polyp from a germline carrier of *POLD1* S478N (9). The top plot for each sample shows variant allele frequency of germline variants, the bottom plot shows the logarithm of coverage for each variant. The red line indicates the position of the constitutional *POLD1* pathogenic mutation.

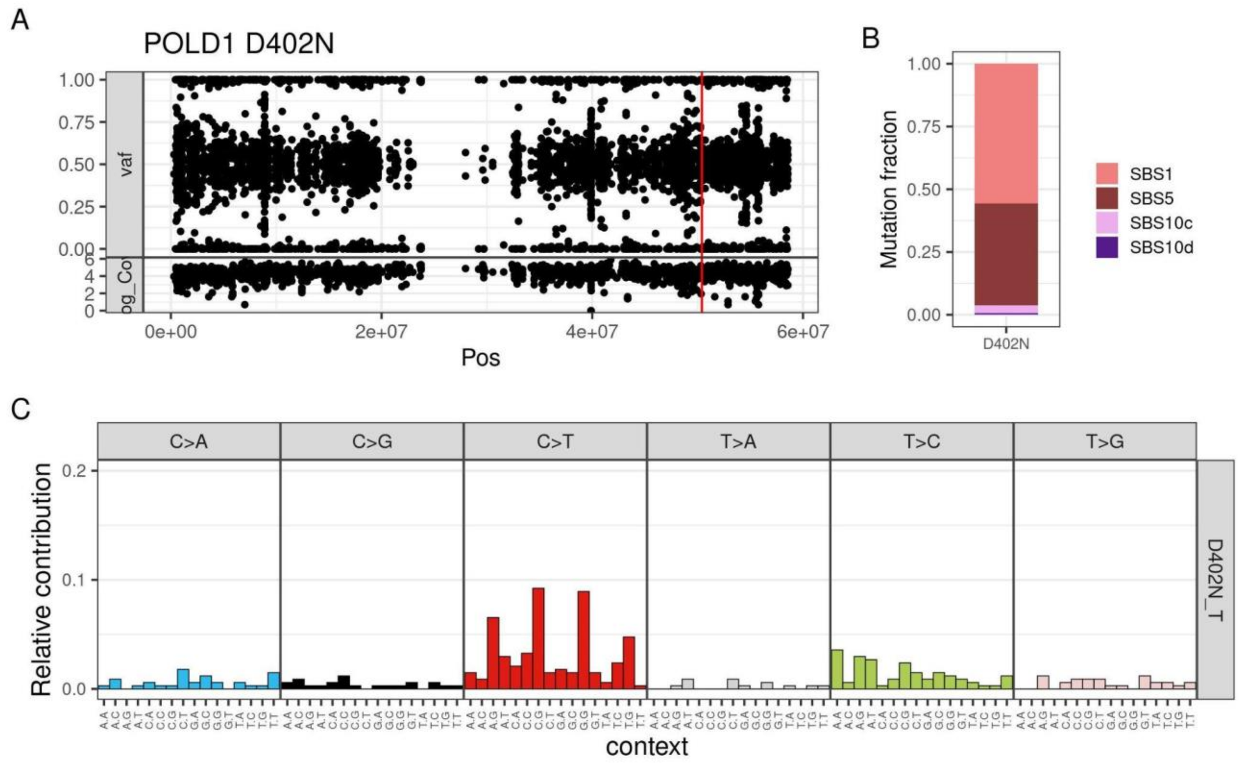

Supplementary Figure S7. Genomic characteristics of tumor from patient with constitutional *POLD1* D402N. **A)** Variant allele frequency of germline variants on chromosome 19. Red line shows the position of the pathogenic variant. **B)** Result of decomposition of mutational spectrum to SBS1, SBS5, SBS10c and SBS10d. **C)** Three-nucleotide mutational spectrum of the tumor.

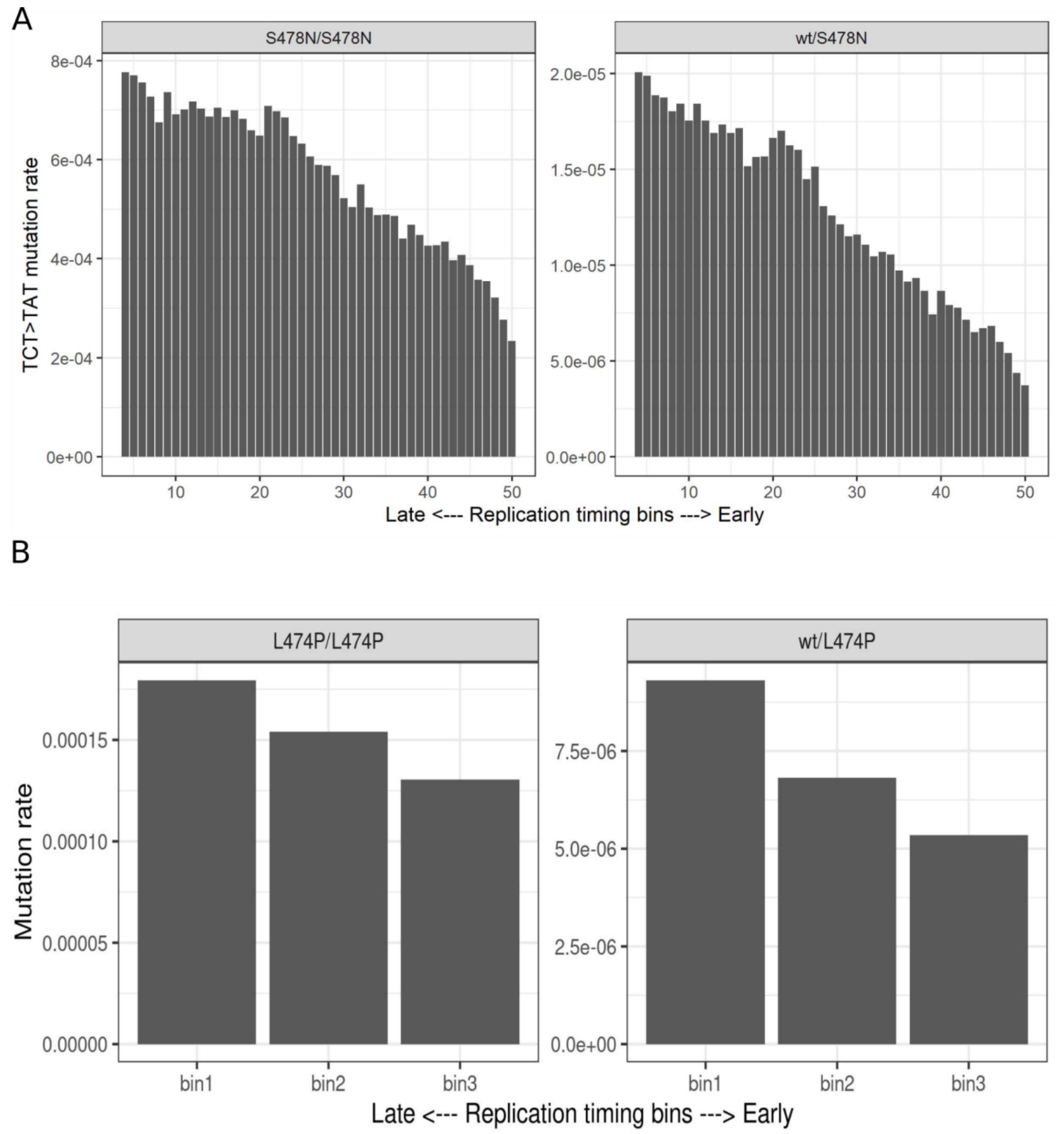

Supplementary Figure S8. Dependence of mutation rate on replication timing in homozygous and heterozygous carriers of **A)** S478N (9) and **B)** L474P (our data).

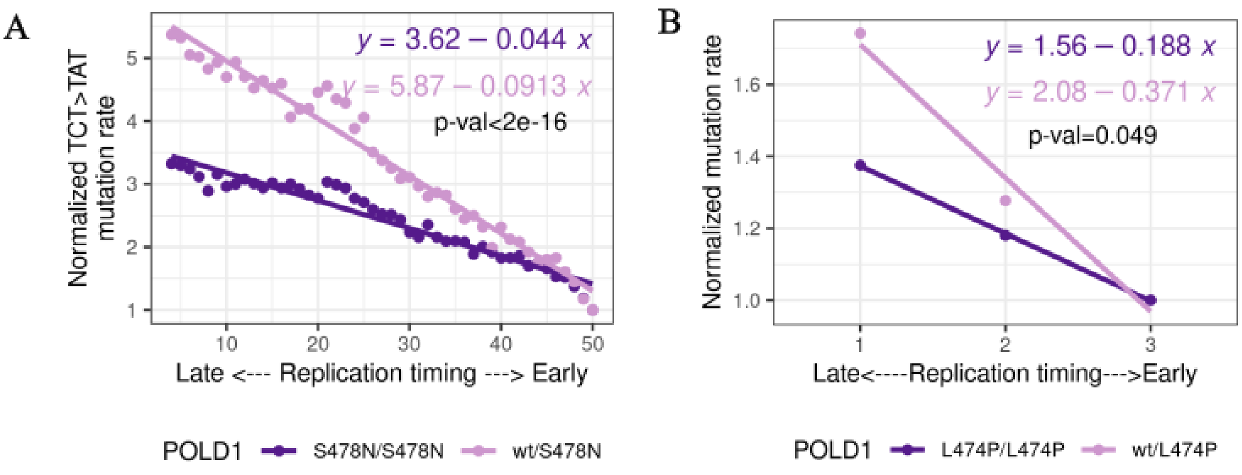

Supplementary Figure S9. Dependence of mutation rate on replication timing in homozygous and heterozygous carriers of **A)** *POLD1* S478N (9) and **B)** *POLD1* L474P (our data).

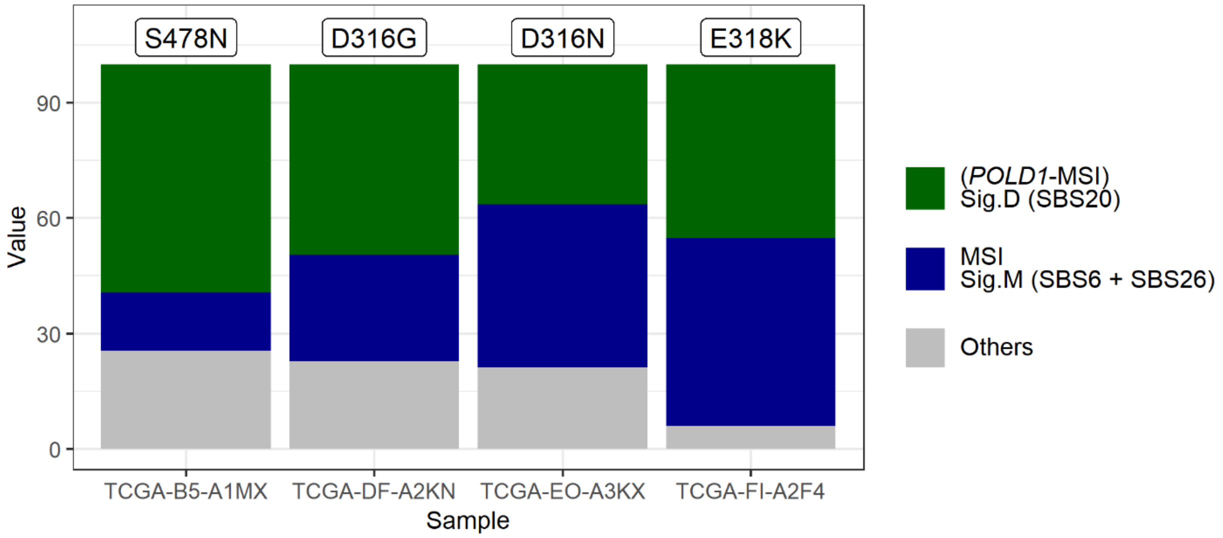

Supplementary Figure S10. Mutational signatures in TCGA UCEC samples with somatic pathogenic variants in *POLD1*. Data obtained from previously published study (25). Sig.D corresponding to simultaneous inactivation of MMR and *POLD1* proofreading is not an additive spectrum of MMR inactivation and POLD1 proofreading inactivation, thus it can be extracted separately from Sig.M with a high level of confidence.

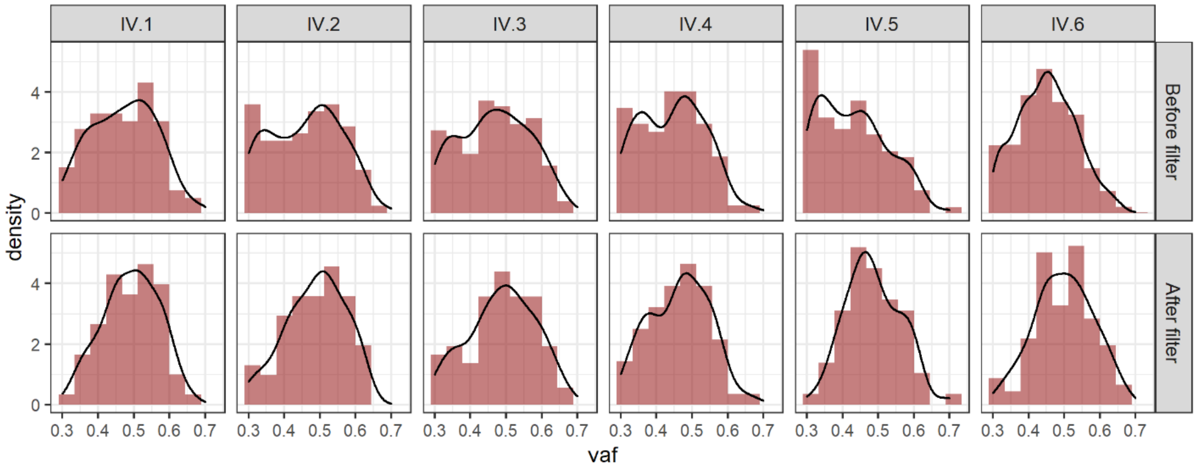

Supplementary Figure S11. Variant allele frequency (VAF) distributions for candidate de novo mutations before and after filtering, using mutations identified in fibroblast colonies.

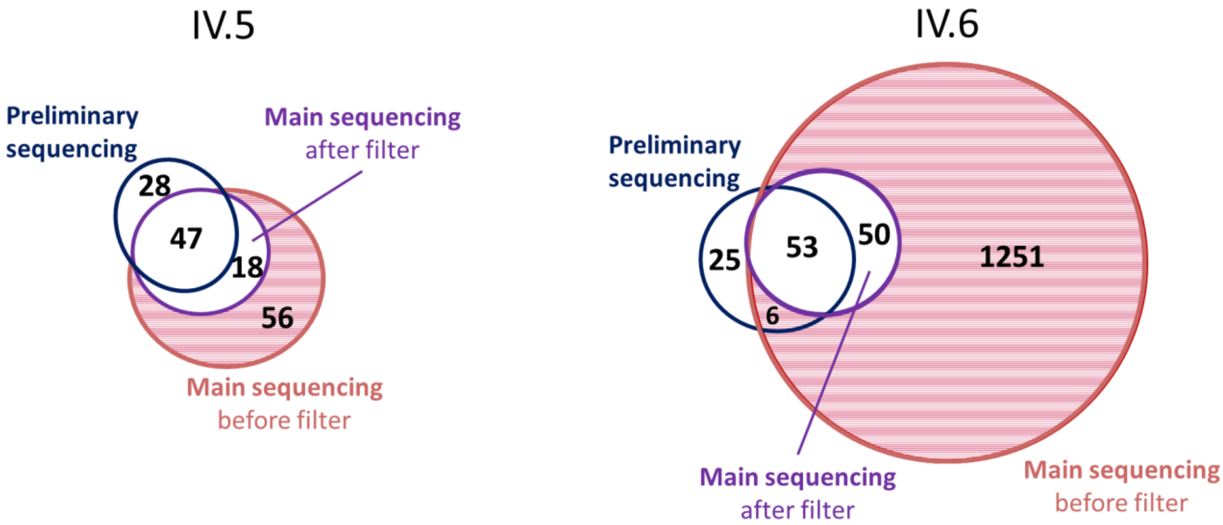

Supplementary Figure S12. Intersection of candidate *de novo* mutations in preliminary sequencing of two trios and in main sequencing before and after the filtering for presence in the fibroblast colony was applied. Dashed red area corresponds to mutations removed by the filter.

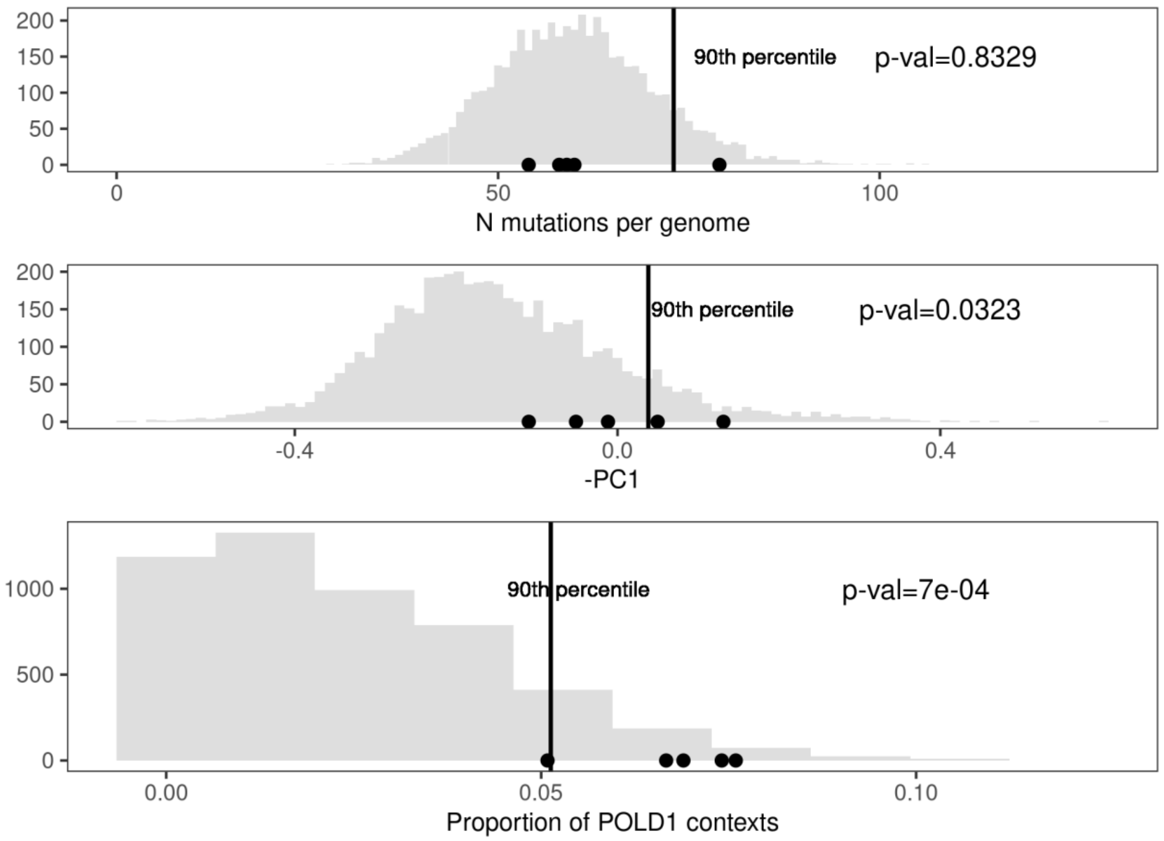

Supplementary Figure S13. Representation of the number of mutations (top), -PC1 values (middle), and proportion of *POLD1*-specific contexts (bottom) in synthetic dataset of wt trios (gray distribution) and trios with 15% of mutations added from spectrum of SBS10c COSMIC signature. The p-value for Kolmogorov-Smirnov test is shown.

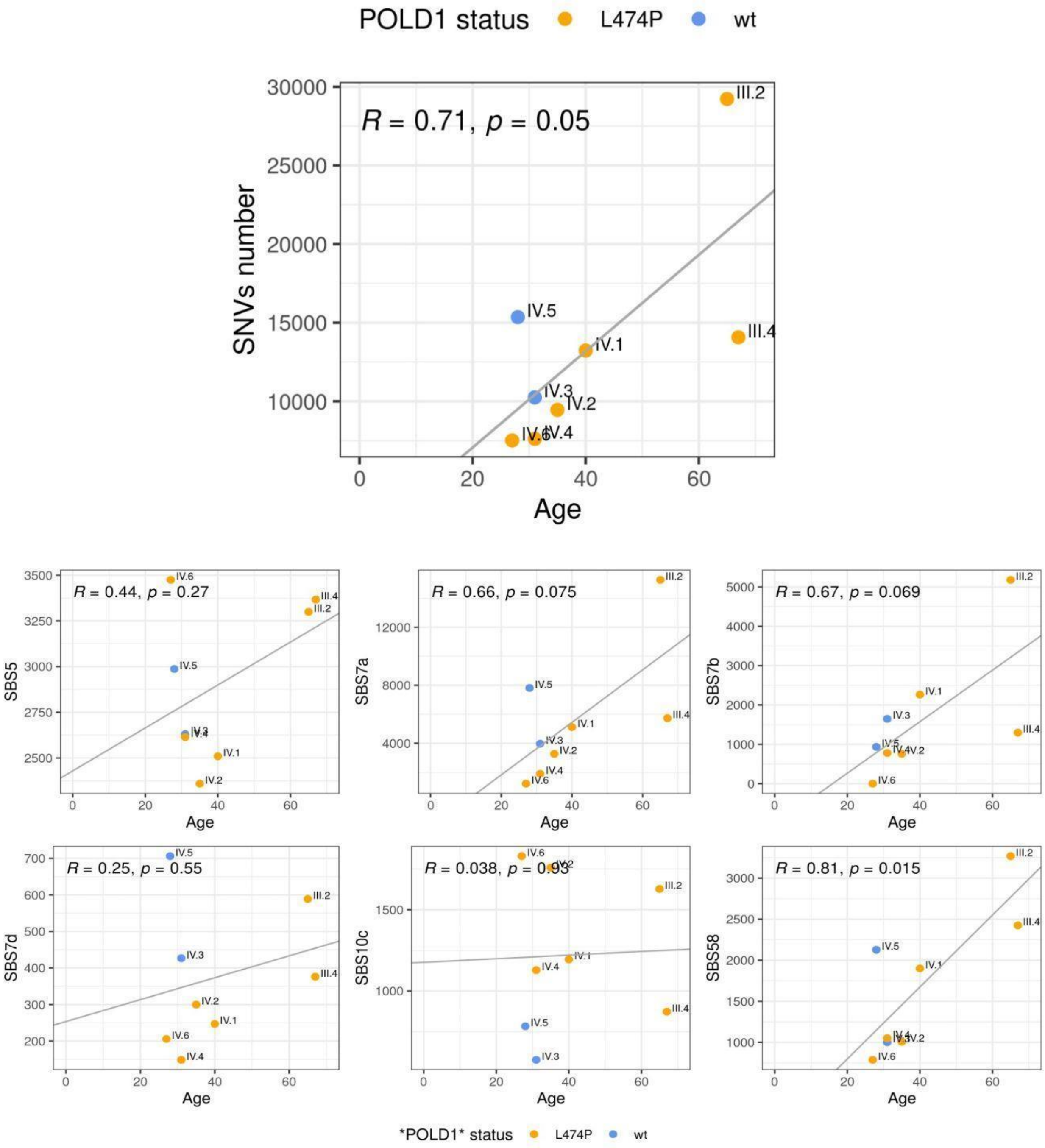

Supplementary Figure S14. Correlation of the number of mutations with individual age at the time of biopsy.

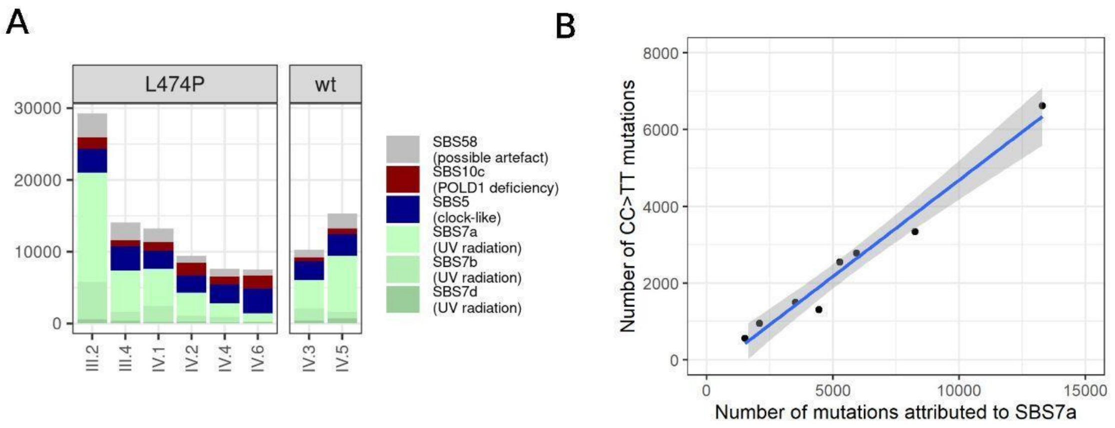

Supplementary Figure S15. Mutations accumulated in skin fibroblasts during life. **A,** COSMIC mutational signatures found in single-cell colonies from skin fibroblasts. The list of signatures was obtained using de novo extraction by SigProfilerExtractor and subsequent refit using SigFit. **B,** Correlation of the number of mutations attributed to SBS7a and number of CC>TT double substitutions in skin fibroblasts colonies.

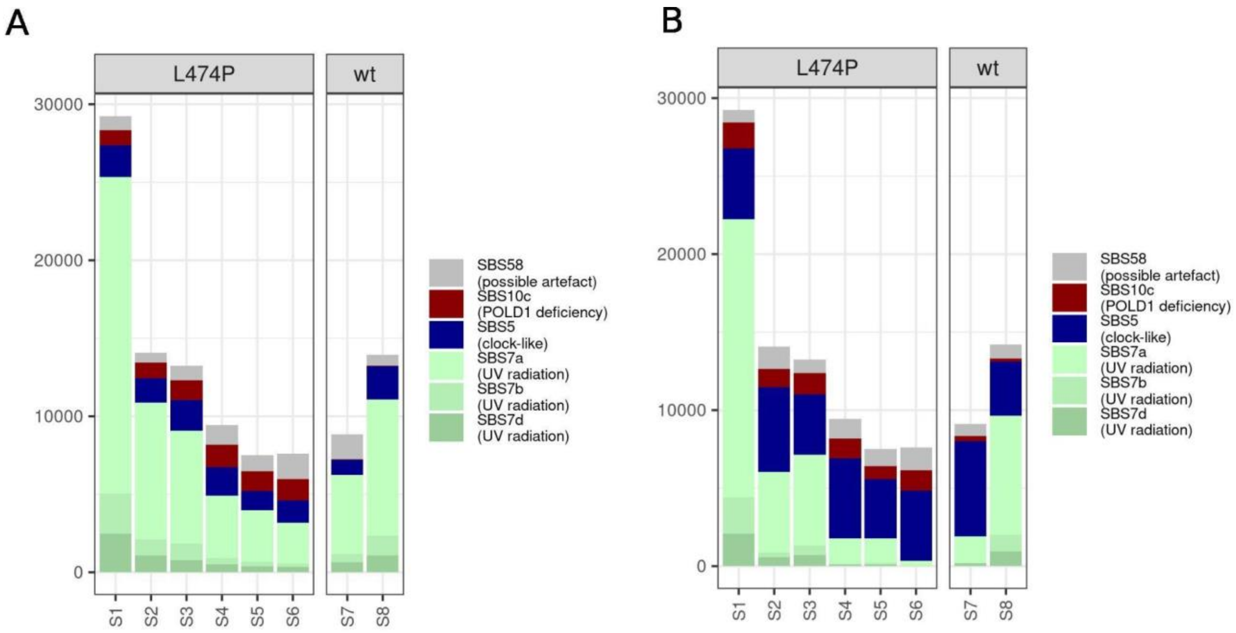

Supplementary Figure S16. Signatures observed in synthetic samples after refitting procedure by SigFit. Synthetic samples were generated with the number of mutations corresponding to real samples. Wildtype samples were generated using SBS5 (500 mutations), SBS58 (1000 mutations) and SBS7a,b,d (other mutations). In L474P samples 1000 mutations from SBS10c were added. **A)** 1000 random mutations representing noise were added. **B)** 4000 random mutations were added.

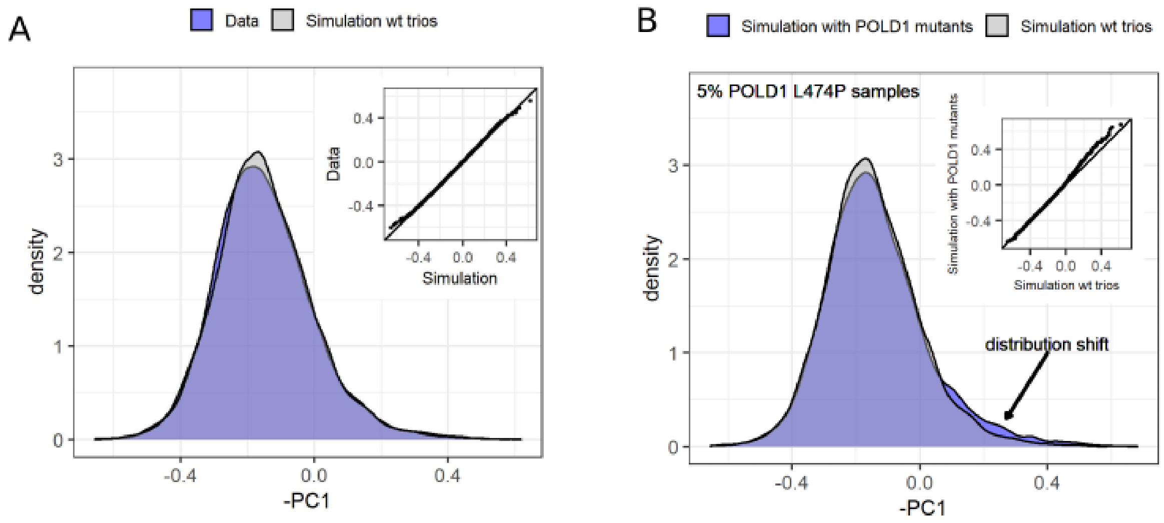

Supplementary Figure S17. A) Distribution of the -PC1 values in published and simulated *de novo* mutation spectra. **B)** Distribution of -PC1 values in simulations of *de novo* mutations with a mixture of offspring of *POLD1* L474P fathers.

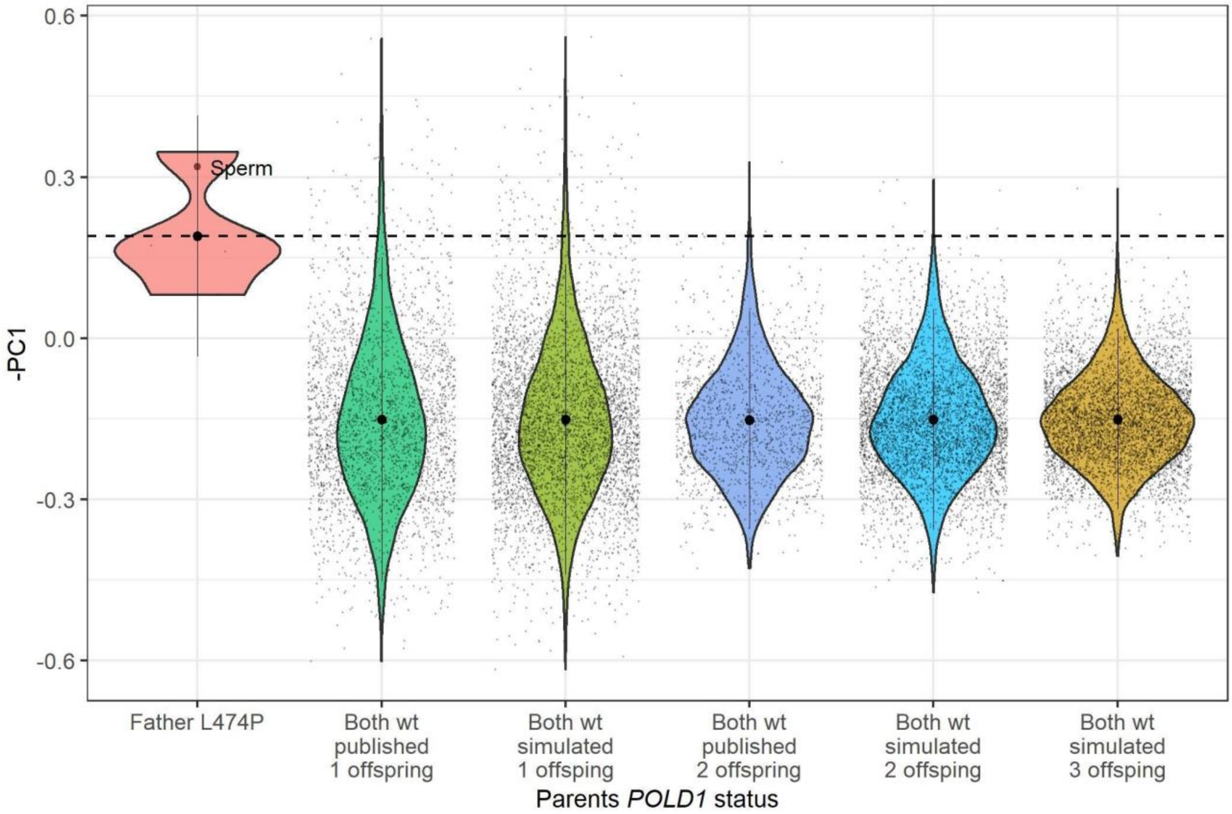

Supplementary Figure S18. Proportion of POLD1-associated contexts in de novo mutations in trios with *POLD1* L474P carrier father and in observed and simulated families with different numbers of offspring. For families with more than one offspring –PC1 value averaged among offspring is shown for each family. Dashed line corresponds to mean –PC1 value in the offspring of fathers harboring *POLD1* L474P.

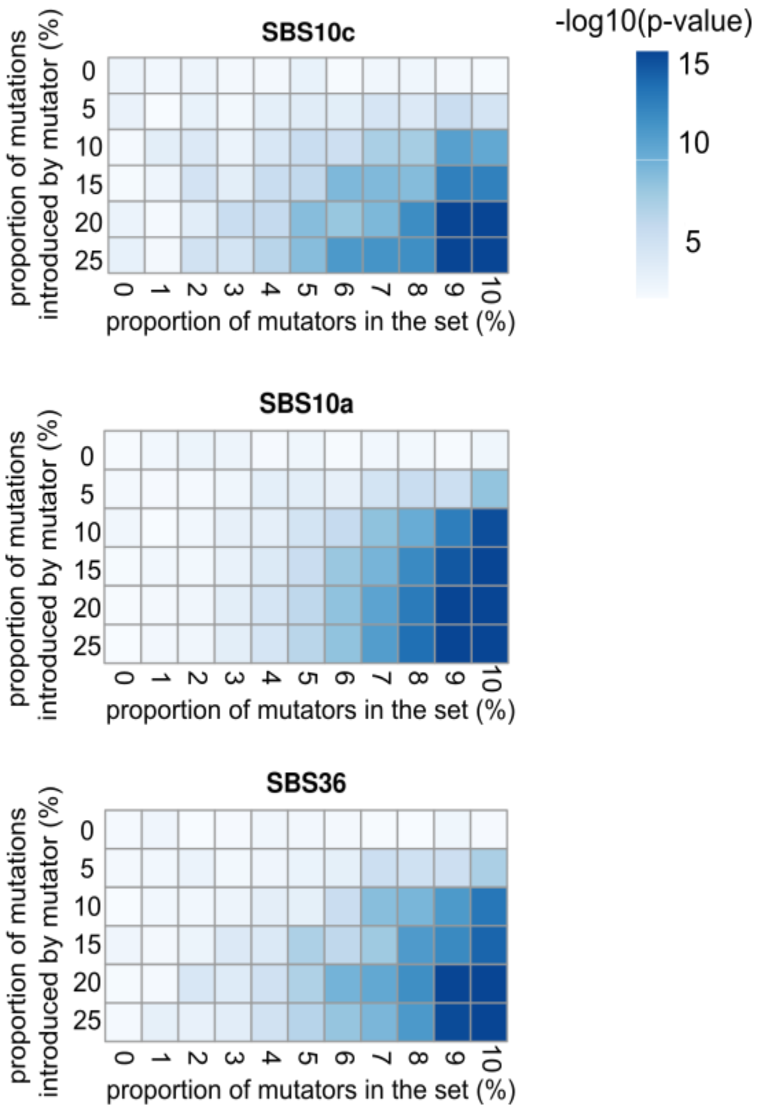

Supplementary Figure S19. Statistical significance (two-sample Kolmogorov-Smirnov test, -log10 p-value) of difference in distributions of cosine similarities to target mutational signature (SBS10c, SBS10a or SBS36) between synthetic wildtype population and synthetic population with presence of mutators. The proportion of mutators in the synthetic population varies between 0% and 10% (x-axis) and the proportion of mutations contributed by additional mutagenic process in the mutators varies between 0% and 25% (y-axis).

SUPPLEMENTARY TABLES

Supplementary Table S1. Clinical and phenotypic characteristics of the family members included in the study.

Individual ID	Sex	*POLD1* status	Tissue sequenced for trio analysis	Single-cell colony from fibroblasts	MA experiment	Diagnosed Polyps/cancer (age of diagnosis)
IV.6	Male	L474P	Fibroblasts	+	-	-
III.2	Male	L474P	Fibroblasts	+	+	Endometrial cancer (58), 9 polyps (since age 48)
III.4	Male	L474P	Blood	+	+	Colon cancer (50), >12 polyps (since age 50)
IV.4	Female	L474P	Fibroblasts	+	+	4 polyps (27)
III.6	Male	L474P	Blood	-	-	Esophageal enteroid metaplasia, ~8 polyps (since age 48), gastric fundic gland polyps (since age 57)
IV.2	Female	L474P	Fibroblasts	+	+	5-10 polyps (since age 25)
IV.1	Female	L474P	Fibroblasts	+	-	Colon cancer (23), esophagus benign tumor* (30)<br>>50 gastric fundic gland polyps (since age 30)
IV.8	Female	wt	Blood	-	-	-
III.7	Female	wt	Blood	-	-	-
III.5	Female	wt	Blood	-	-	-
IV.5	Male	wt	Buccal swab	+	+	-
IV.3	Male	wt	Fibroblasts	+	+	-
III.1	Female	wt	Blood	-	-	-
III.3	Female	wt	Blood	-	-	-
III.8	Male	wt	Blood	-	-	-
IV.9	Female	wt	Buccal swab	-	-	-

*compatible with GIST by echoendoscopy.
Abbreviations: MA, mutation accumulation.

Supplementary Table S2. Characteristics of the fibroblast cultures in the mutation accumulation experiment.

Sample	Total # of passages	# of passages from single cell isolation until DNA sequencing	passage doubling rate	*POLD1* status
III.2	77	34	1.62	L474P
IV.2	87	46	1.58	L474P
IV.3	67	35	1.49	wt
III.4	60	38	NA	L474P
IV.4	63	33	2.14	L474P
IV.5	54	33	2.07	wt

Supplementary Table S3. Statistical significance of presence of SBS10c signature in mutations accumulated during the experiment calculated using mSigAct package.

Sample	*POLD1* status	p-value
IV.6	L474P	1.430436e-24***
III.2	L474P	1.817e-34***
IV.5	wt	0.01964926*
III.4	L474P	8.094666e-24**
IV.3	wt	0.8072493
IV.4	L474P	3.63662e-55**

Supplementary Table S4. Probability of LOH in the studied samples.

POLD1 variant	Sites with cnLOH	Sites with LOH (cnLOH or deletion)	Target sites	cnLOH probability	LOH probability
L474P	3036874	3603390	2628131683	$1.16*10^{-3}$	$1.37*10^{-3}$
D316H	143778197	266663050	2587827238	$5.56*10^{-2}$	$1.03*10^{-1}$
S478N	661469	18389853	2615521263	$2.53*10^{-4}$	$7.03*10^{-3}$
Simultaneous probability				$1.62*10^{-8}$	$9.92*10^{-7}$

Supplementary Table S5. MMR status of tumors with constitutional *POLD1* pathogenic variants.

***POLD1* nucleotide substitution**	***POLD1* amino acid substitution**	**Cancer type**	**Reference**	**MSI/MSS status**
c.947A>G	D316G	Colorectal	Bellido et al. 2016	MSS
c.947A>G	D316G	Endometrial	Bellido et al. 2016	MSS
c.1433G>A	S478N	Colorectal	Palles et al. 2013 and 2021	MSS (tumor, 1 AP)
c.1433G>A	S478N	Adenoma	Palles et al. 2013 and 2021	MSS (5 AP)
c.1433G>A	S478N	Endometrial	Palles et al. 2013 and 2021	MSS
c.1433G>A	S478N	Colorectal	Ito et al. 2020	Normal MMR protein expression
c.1421T>C	L474P	Colorectal	Valle et al. 2014	MSS
c.1421T>C	L474P	Colorectal	Bellido et al. 2016	MSS
c.1421T>C	L474P	Colorectal	Ferrer-Avargues et al. 2017	MSI
c.1421T>C	L474P	Colorectal	Ferrer-Avargues et al. 2017	MSS

Abbreviations: AP, adenomatous polyp; MMR, DNA mismatch repair; MSI, microsatellite instability (MMR deficiency); MSS, microsatellite stability (MMR proficiency)

SUPPLEMENTARY REFERENCES

1. Li H. Aligning sequence reads, clone sequences and assembly contigs with BWA-MEM. arXiv:1303.3997v2; 2013.
2. Islam SMA, Díaz-Gay M, Wu Y, Barnes M, Vangara R, Bergstrom EN, et al. Uncovering novel mutational signatures by de novo extraction with SigProfilerExtractor. Cell Genom. 2022;2(11):None.
3. Gori K, Baez-Ortega A. sigfit: flexible Bayesian inference of mutational signatures. bioRxiv. 2020:372896.
4. Ng AWT, Poon SL, Huang MN, Lim JQ, Boot A, Yu W, et al. Aristolochic acids and their derivatives are widely implicated in liver cancers in Taiwan and throughout Asia. Sci Transl Med. 2017;9(412).
5. Manders F, Brandsma AM, de Kanter J, Verheul M, Oka R, van Roosmalen MJ, et al. MutationalPatterns: the one stop shop for the analysis of mutational processes. BMC Genomics. 2022;23(1):134.
6. Bhagwate AV, Liu Y, Winham SJ, McDonough SJ, Stallings-Mann ML, Heinzen EP, et al. Bioinformatics and DNA-extraction strategies to reliably detect genetic variants from FFPE breast tissue samples. BMC Genomics. 2019;20(1):689.
7. Ryba T, Hiratani I, Lu J, Itoh M, Kulik M, Zhang J, et al. Evolutionarily conserved replication timing profiles predict long-range chromatin interactions and distinguish closely related cell types. Genome Res. 2010;20(6):761-70.
8. Pope BD, Ryba T, Dileep V, Yue F, Wu W, Denas O, et al. Topologically associating domains are

stable units of replication-timing regulation. Nature. 2014;515(7527):402-5.
9. Robinson PS, Coorens THH, Palles C, Mitchell E, Abascal F, Olafsson S, et al. Increased somatic mutation burdens in normal human cells due to defective DNA polymerases. Nat Genet. 2021;53(10):1434-42.
10. Talevich E, Shain AH, Botton T, Bastian BC. CNVkit: Genome-Wide Copy Number Detection and Visualization from Targeted DNA Sequencing. PLoS Comput Biol. 2016;12(4):e1004873.
11. Richards S, Aziz N, Bale S, Bick D, Das S, Gastier-Foster J, et al. Standards and guidelines for the interpretation of sequence variants: a joint consensus recommendation of the American College of Medical Genetics and Genomics and the Association for Molecular Pathology. Genet Med. 2015;17(5):405-24.
12. Mur P, Viana-Errasti J, García-Mulero S, Magraner-Pardo L, Muñoz IG, Pons T, et al. Recommendations for the classification of germline variants in the exonuclease domain of POLE and POLD1. Genome Med. 2023;15(1):85.
13. Valle L, Hernández-Illán E, Bellido F, Aiza G, Castillejo A, Castillejo MI, et al. New insights into POLE and POLD1 germline mutations in familial colorectal cancer and polyposis. Hum Mol Genet. 2014;23(13):3506-12.
14. Bellido F, Pineda M, Aiza G, Valdés-Mas R, Navarro M, Puente DA, et al. POLE and POLD1 mutations in 529 kindred with familial colorectal cancer and/or polyposis: review of reported cases and recommendations for genetic testing and surveillance. Genet Med. 2016;18(4):325-32.
15. Ferrer-Avargues R, Díez-Obrero V, Martín-Tomás E, Hernández-Illán E, Castillejo MI, Codoñer-Alejos A, et al. Characterization of a novel POLD1 missense founder mutation in a Spanish population. J Gene Med. 2017;19(4).
16. Palles C, Martin L, Domingo E, Chegwidden L, McGuire J, Cuthill V, et al. The clinical features of polymerase proof-reading associated polyposis (PPAP) and recommendations for patient management. Fam Cancer. 2022;21(2):197-209.
17. Ioannidis NM, Rothstein JH, Pejaver V, Middha S, McDonnell SK, Baheti S, et al. REVEL: An Ensemble Method for Predicting the Pathogenicity of Rare Missense Variants. Am J Hum Genet. 2016;99(4):877-85.
18. Murphy K, Darmawan H, Schultz A, Fidalgo da Silva E, Reha-Krantz LJ. A method to select for mutator DNA polymerase deltas in Saccharomyces cerevisiae. Genome. 2006;49(4):403-10.
19. Saini N, Giacobone CK, Klimczak LJ, Papas BN, Burkholder AB, Li JL, et al. UV-exposure, endogenous DNA damage, and DNA replication errors shape the spectra of genome changes in human skin. PLoS Genet. 2021;17(1):e1009302.
20. Tang J, Fewings E, Chang D, Zeng H, Liu S, Jorapur A, et al. The genomic landscapes of individual melanocytes from human skin. Nature. 2020;586(7830):600-5.
21. Kaplanis J, Ide B, Sanghvi R, Neville M, Danecek P, Coorens T, et al. Genetic and chemotherapeutic influences on germline hypermutation. Nature. 2022;605(7910):503-8.

22. Mur P, García-Mulero S, Del Valle J, Magraner-Pardo L, Vidal A, Pineda M, et al. Role of POLE and POLD1 in familial cancer. Genet Med. 2020;22(12):2089-100.
23. Halldorsson BV, Palsson G, Stefansson OA, Jonsson H, Hardarson MT, Eggertsson HP, et al. Characterizing mutagenic effects of recombination through a sequence-level genetic map. Science. 2019;363(6425).
24. An JY, Lin K, Zhu L, Werling DM, Dong S, Brand H, et al. Genome-wide de novo risk score implicates promoter variation in autism spectrum disorder. Science. 2018;362(6420).
25. Haradhvala NJ, Polak P, Stojanov P, Covington KR, Shinbrot E, Hess JM, et al. Mutational Strand Asymmetries in Cancer Genomes Reveal Mechanisms of DNA Damage and Repair. Cell. 2016;164(3):538-49.
